# Multicomponent synthesis of spiropyrrolidine analogues derived from vinylindole/indazole by a 1,3-dipolar cycloaddition reaction

**DOI:** 10.3762/bjoc.12.288

**Published:** 2016-12-29

**Authors:** Manjunatha Narayanarao, Lokesh Koodlur, Vijayakumar G Revanasiddappa, Subramanya Gopal, Susmita Kamila

**Affiliations:** 1 East Point College of Engineering and Technology, VisvesvarayaTechnological University, Aavalahalli, Bangalore-560 049, India; 2 Department of Studies and Research in Chemistry, Vijayanagara Sri Krishnadevaraya University, Bellary-583105, India; 3 Department of Chemistry, University College of Science, Tumkur University, BH Road, Tumakuru-572103 India

**Keywords:** L-proline, ninhydrin, sarcosine, spiropyrrolidine, 5-vinylindazole, 5-vinylindole

## Abstract

A new series of spiropyrrolidine compounds containing indole/indazole moieties as side chains have been accomplished via a one-pot multicomponent synthesis. The method uses the 1,3-dipolar cycloaddition reaction between *N*-alkylvinylindole/indazole and azomethine ylides, prepared in situ from cyclic/acyclic amino acids. The 1,3-dipolar cycloaddition proceeds efficiently under thermal conditions to afford the regio- and stereospecific cyclic adducts.

## Introduction

The [3 + 2] cycloaddition between azomethine ylides and olefins/acetylene as dipolarophiles is an important reaction to access a number of novel heterocyclic spiro scaffolds of biological importance [1–2]. The regio- and stereoselective construction of spiro compounds by utilizing the 1,3-dipolar cycloaddition reaction of azomethine ylides has been reported [3–6]. However, unlike the previous reports, the present method is concise and facilitates the one-pot synthesis of multifunctionalized spiranes. The earlier works reveal the use of different reaction conditions to prepare functionalized spiroindane, spiropyrolidine, spiropyrrolizidine and spirooxindole derivatives. Among them, the 1,3-dipolar cycloaddition based route is preferred due to multiple advantages [7–10].

Indole and indazolyl subunits are central to numerous alkaloids and are constituents of many classes of compounds that display a wide range of bioactivities such as antimycobacterial, anti-inflammatory, antihypertensive, anticancer, antidepressant, antidiabetic, antimalarial, antitubercular, anticonvulsant and cardiovascular activities [11–16].

As part of our interest in the exploration of 1,3-dipolar cycloaddition reactions [17] for the synthesis of polycyclic molecules, *N*-alkylated vinylindoles are applied as dipolarophiles in view of the challenges that still pose in the construction of functionalized spiro compounds. We report herein the facile synthesis of novel spiropyrrolidine compounds through a one-pot three-component reaction involving N-substituted vinylindole/indazole, ninhydrin and sarcosine/L-proline. The present multicomponent reaction (MCR) leads to five-membered spiropyrrolidine derivatives **7** and **8** substituted with indole or indazole moieties, respectively. The synthesis being described is the first on the usage of *N*-alkylated vinylindole or indazole derivatives.

## Results and Discussion

**Scheme 1:**
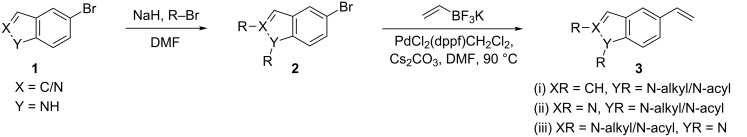
Synthesis of *N*-alkyl vinylindoles and *N*-alkyl vinylindazoles (**3**).

Our studies started with the synthesis of 5-bromo-*N*-alkylindole and indazole derivatives **2** that can be obtained from 5-bromoindoles/indazoles (**1**). The latter compounds were synthesized according to procedures described in the literature [18–20]. The regioisomers of *N*-alkylated 5-bromoindazoles were isolated by column chromatography using a hexane–ethyl acetate mixture. The resulting 5-bromo-*N*-alkyl derivatives **2** were then treated with potassium vinyltrifluoroborate and PdCl_2_(dppf)CH_2_Cl_2_ in DMF at 90 °C to afford the vinyl-substituted products **3** in excellent yields (Scheme 1) [21–22].

**Scheme 2:**
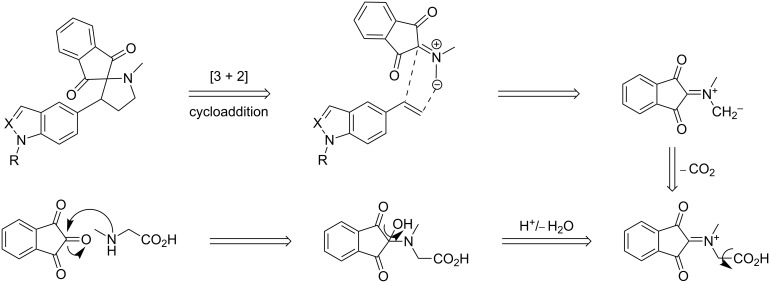
Retrosynthetic strategy used for the synthesis of **7** and **8**.

In the next step, the one-pot three component reaction was carried out by reacting *N*-alkylvinyl products **3** with azomethine ylide, generated in situ through decarboxylative condensation of ninhydrin (**4**) and sarcosine (**5**). The 1,3-dipolar cycloaddition of the ylide with the olefin **3** yielded spiropyrrolidines **7** with regiospecificity (Table 1, entries 1–4). The formation of the azomethine ylide intermediate and a plausible reaction pathway for the formation of the spiranes is depicted following the retrosynthetic strategy in Scheme 2.

**Table 1:** Synthesis of spiropyrrolidine compounds **7a–k** and **8a–k**.^a^

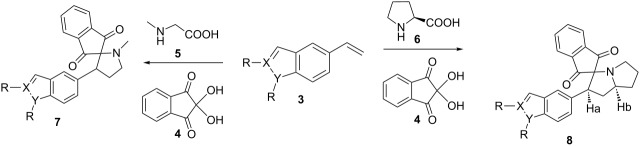				

Entry	X–R	Y–R	Product	Yield^b^ (%)

1	C–H	N–CH_3_	**7a**	90
2	C–H	N–CH_2_CH(CH_3_)_2_	**7b**	93
3	C–H	N–COCH_3_	**7c**	87
4	C–H	N–CH_2_C_6_H_5_	**7d**	91
5	N	N–CH_3_	**7e**	85
6	N	N–CH_2_CH(CH_3_)_2_	**7f**	82
7	N	N–COCH_3_	**7g**	78
8	N	N–CH_2_C_6_H_5_	**7h**	79
9	N–CH_3_	N	**7i**	70
10	N–CH_2_CH(CH_3_)_2_	N	**7j**	65
11	N–CH_2_C_6_H_5_	N	**7k**	60
12	C–H	N–CH_3_	**8a**	86
13	C–H	N–CH_2_CH(CH_3_)_2_	**8b**	80
14	C–H	N–COCH_3_	**8c**	83
15	C–H	N–CH_2_C_6_H_5_	**8d**	85
16	N	N–CH_3_	**8e**	86
17	N	N–CH_2_CH(CH_3_)_2_	**8f**	68
18	N	N–COCH_3_	**8g**	70
19	N	N–CH_2_C_6_H_5_	**8h**	82
20	N–CH_3_	N	**8i**	68
21	N–CH_2_CH(CH_3_)_2_	N	**8j**	68
22	N–CH_2_C_6_H_5_	N	**8k**	68

^a^Reaction conditions: **3** (1 mmol, 1 equiv), **4** (1.2 mmol, 1.2 equiv), **5**/**6** (1.2 mmol, 1.2 equiv), methanol (5 mL), 60 °C, 30 min. ^b^Isolated yield.

The applicability of the cycloaddition reaction was explored first for indole derivatives **7a–d** and then extended to the synthesis of indazole analogues **7e–k** under similar reaction conditions. Based on the same logic of vinylindole, regioisomers of *N*-alkylvinylindazole were considered. So, this 1,3-dipolar cycloaddition reaction could be well applied to both derivatives which afforded a range of differentially substituted final products in yields ranging from 60 to 93% (Table 1, entries 1–11). Unfortunately the product **7** with X–R = NCOCH_3_, Y = N could not be synthesized due to low amounts of the required regioisomer of the *N*-acylated indazole. In both cases of vinylic indole and indazoles, we have shown that the cyclic adducts were favored with dipoles and highly reactive dipolarophiles (vinylindole and -indazole). The increase in the reactivity could be attributed to the presence of electron-donating groups on the dipolarophile.

**Figure 1:**
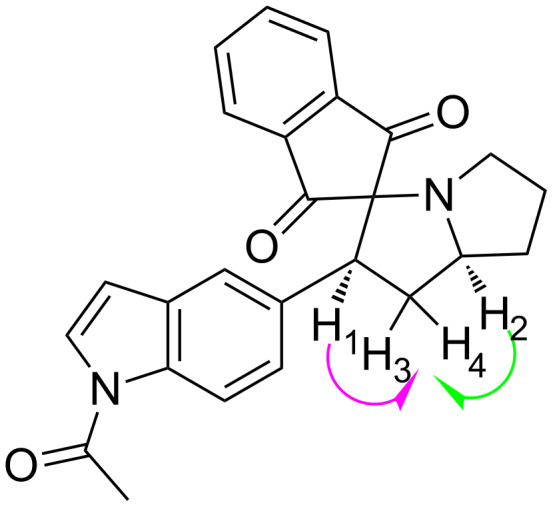
NOE interactions in compound **8c** supporting the stereochemical assignments for **8a–k**. H_1_ and H_2_ on the asymmetric carbon atoms were found to be syn oriented.

Encouraged by the success with the acyclic amino acid sarcosine (**5**), the reaction was extended to the cyclic amino acid L-proline (**6**) which was reacted with **3** under the optimized reaction conditions. The corresponding stereo- and regiospecific isomers of spiropyrrolidines **8** were obtained in good yields **(**Table 1, entries 12–22). The stereochemical assignments for compounds **8** were supported by 2D NMR data which showed NOE interactions between H_1_ and H_2_ on the asymmetric carbon atoms correlated to the *syn*-configuration (Figure 1). Upon irradiation of H_1_ the NOE enhancement was observed for H_3_ and H_4_ and the same was observed by irradiation of the ring-junction proton H_2_. The regioselectivity of cyclic adducts could be explained by secondary orbital interaction as described in earlier reports [4,23]. All compounds were comprehensively characterized by spectroscopic methods (IR, ^1^H, ^13^C NMR and LC–MS). The general procedure for the synthesis of **7a–k** and **8a–k** including the spectral characterization of the compounds are provided in Supporting Information File 1.

**Figure 2:**
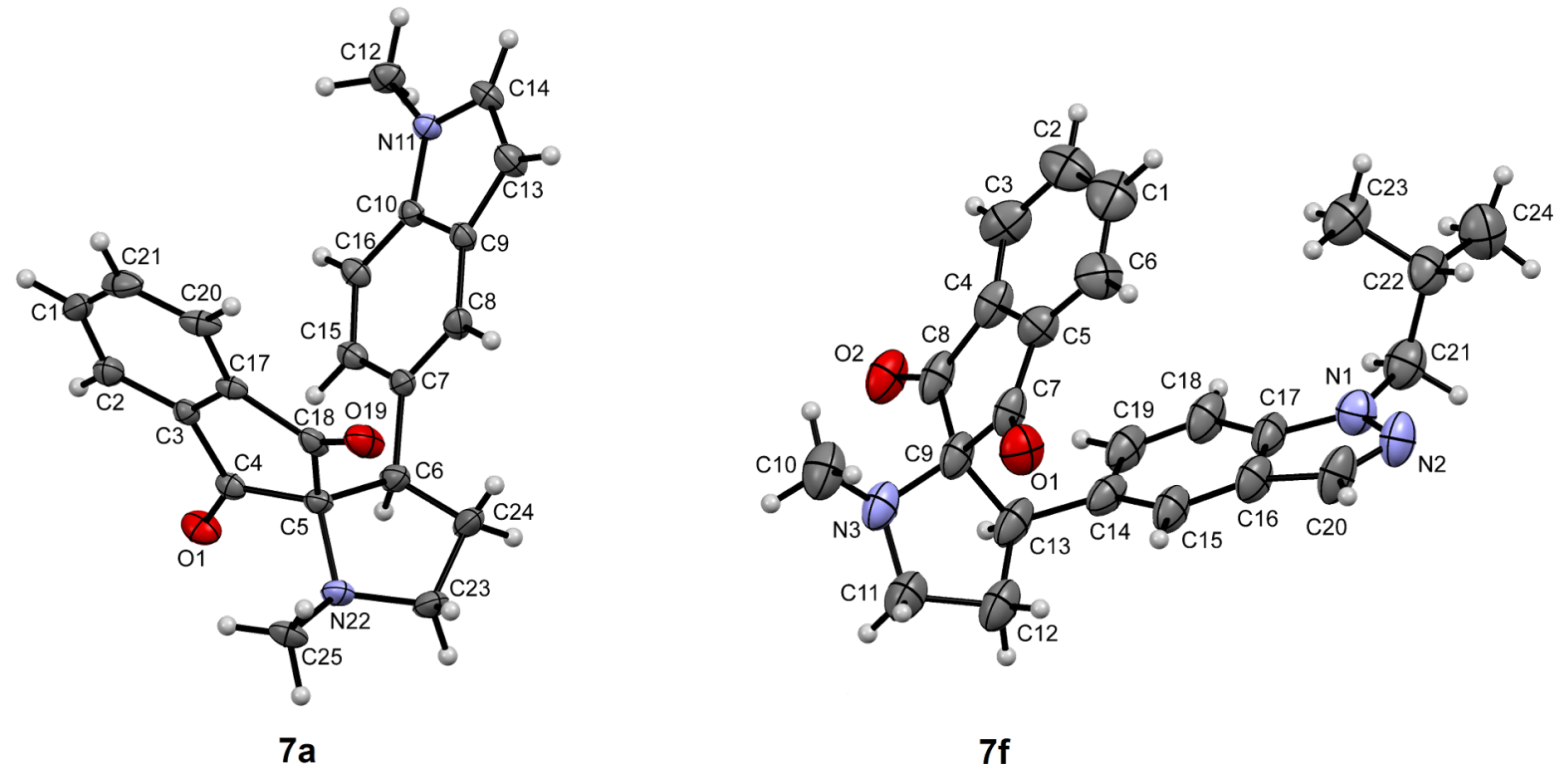
ORTEP representation of compounds **7a** and **7f** obtained by single crystal XRD study.

The ^1^H NMR spectrum of compound **7a** (X–R = CH, Y–R = N–CH_3_) proved the formation of the desired product which showed a characteristic doublet of doublets for the H_1_ proton at δ 3.99 ppm. The CH_2_ group adjacent to the pyrrolidine nitrogen appeared as a triplet of doublets (δ 3.42 ppm) and multiplet (δ 3.59 ppm), and the N–CH_3_ of the pyrrolidine ring as a singlet at δ 2.36 ppm. Further, the structures of **7a** and **7f** were confirmed by single crystal X-ray diffraction studies and the corresponding ORTEP representations are given in Figure 2.

## Conclusion

In conclusion, the present work explores the use of 1,3-dipolar cycloaddition reactions for the regio- and stereospecific synthesis of functionalized spiropyrrolidines through a one-pot multicomponent reaction. This new route involving a vinyl framework gives rise to a wide array of novel compounds that could find use in different areas of chemistry. The advantages of the synthesis described are its ease of execution, short reaction times and diversity of the products synthesized.

## Supporting Information

File 1 Experimental and characterization data of all new compounds.
