# Supplementary material for: Multicomponent synthesis of spiropyrrolidine analogues derived from vinylindole/indazole by a 1,3-dipolar cycloaddition reaction
Source: Beilstein J Org Chem. 2016 Dec 29;12:2893–7. doi: 10.3762/bjoc.12.288 (PMC5238586; doi:10.3762/bjoc.12.288)
Supplement: File 1 — Experimental and characterization data of all new compounds. [file Beilstein_J_Org_Chem-12-2893-s001.pdf]

Supporting Information

for

Multicomponent synthesis of spiropyrrolidine analogues derived from vinylindole/indazole by a 1,3-dipolar cycloaddition reaction

Manjunatha Narayanarao[1], Lokesh Koodlur[2], Vijayakumar G. Revanasiddappa[3], Subramanya Gopal[2], Susmita Kamila[*,1]

Address: [1]East Point College of Engineering and Technology, VisvesvarayaTechnological University, Aavalahalli, Bangalore-560 049, India, [2]Department of Studies and Research in Chemistry, Vijayanagara Sri Krishnadevaraya University, Bellary-583105, India and [3]Department of Chemistry, University College of Science, Tumkur University, BH Road, Tumakuru-572103 India

Email: Susmita Kamila - sushkam@yahoo.co.in

*Corresponding author

Experimental and characterization data of all new compounds

General Information

All reagents and solvents were pure and analytical grade purchased from commercial sources and were used without further purification, if not stated otherwise. Melting points were measured using Buchi B-540 apparatus and were uncorrected. ^{1}H-NMR and ^{13}C NMR spectra were recorded on Bruker DPX 400 MHz FT-NMR spectrometer. Chemical shifts were expressed in δ (ppm) units relative to tetramethylsilane (TMS) signal as internal reference. Mass spectra were recorded on ESQUIRE 3000 Mass Spectrometer. Column chromatography was performed on silica gel (60-120 mesh) using ethyl acetate and hexane as eluent. All reactions were carried out in an over-dried Schlenk tube equipped with a magnetic stir bar under N2 atmosphere.

Analytical data for compounds 2a–k

5-Bromo *N*-alkyl indole and indazole derivatives 2; General Procedure [1-4]

NaH (6.1 mmol, 1.2 equiv) was slowly added to a solution of 5-bromoindole/indazole (5.1 mmol, 1 equiv) in DMF (10 mL) at 0 °C. The mixture was stirred at 0 °C for 10 min then the alkyl halide (6.1 mmol, 1.2 equiv) was added at the same temperature. The reaction mixture was continued to stir at rt for 2 h. When the reaction was complete [TLC (EtOAc/hexane 1:5)], the mixture was extracted with EtOAc (2 × 20 mL) and washed with water (2 × 20 mL). The organic layer was separated, dried over anhydrous Na_2SO_4 and the solvent removed under reduced pressure. The residue obtained was purified by flash column chromatography [silica gel (230–400 mesh; Merck), EtOAc/hexane 1:5].

5-Bromo-*N*-acetylindole or indazole derivatives 2c, 2g; General Procedure [5-6]

Triethylamine (3.8 mmol, 1.5 equiv), acetic anhydride (9.6 mmol, 3.8 equiv) and DMAP (0.5 mmol, 0.2 equiv) were added at rt to a solution of 5-bromo-1*H*-indole (2.5 mmol, 1 equiv) in 1,2 dichloroethane (10 mL). The solution was heated to 60 °C for 8 h. When the reaction was complete [TLC (EtOAc/hexane 2:5)], the mixture was extracted with EtOAc (2 × 20 mL) and washed with water (2 × 20 mL). The organic layer was separated, dried over anhydrous Na_2SO_4, filtered and the solvent removed under reduced pressure. The residue was purified by flash column chromatography [silica gel (230–400 mesh; Merck), EtOAc/hexane 2:5].

5-Bromo-1-methyl-1*H*-indole (2a) [1]

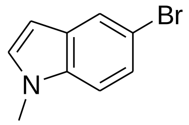

Colorless viscous liquid; yield 0.150g (0.71 mmol, 81%). ^{1}H NMR (300 MHz, DMSO-d6): δ = 7.73 (1 H, d, ^{3}J = 1.80 Hz, CH), 7.37-7.43 (2 H, m, 2 CH), 7.23-7.27 (1 H, m, CH), 6.41 (1 H, d, ^{3}J = 3.00 Hz, 1H), 3.78 (3 H, s, Me). LCMS: *m/z* (%) = 210.0 [M+1]. Anal. Calcd for C_9H_8BrN (208.98): C, 51.46; H, 3.84; Br, 38.04; N, 6.67. Found: C, 51.52; H, 3.89; Br, 38.15; N, 6.70.

5-Bromo-1-isobutyl-1*H*-indole (2b) [2]

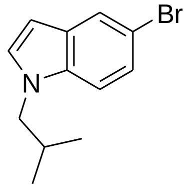

Colorless viscous liquid; yield 0.18g (0.71 mmol, 80%). ^{1}H NMR(400 MHz, DMSO-d6): δ = 7.72 (d, ^{3}J = 2.40 Hz, 1 H), 7.40-7.49 (m, 2 H), 7.22 (dd, ^{3}J = 14.40, Hz, 1 H), 7.42 (d, ^{3}J = 4.00 Hz, 1 H), 3.98 (d, ^{3}J = 9.60 Hz, 2 H), 2.05-2.14 (m, 1 H), 0.82 (dd, ^{3}J = 8.80, Hz, 6 H). LCMS: *m/z* (%) = 252.2 [M+1]. Anal. Calcd for $C_{12}H_{14}BrN$ (251.03): C, 57.16; H, 5.60; Br, 31.69; N, 5.55. Found: C, 57.25; H, 5.63; Br, 31.70, N, 5.59.

1-(5-Bromo-1*H*-indol-1-yl)ethanone (2c) [5]

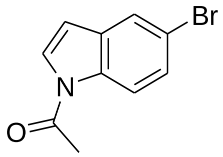

Off white solid; yield 0.22g (0.92 mmol, 66%). ^{1}H NMR(400 MHz, DMSO-d6): δ = 8.27 (1 H, d, ^{3}J = 8.80 Hz, CH), 7.92 (1 H, d, ^{3}J = 3.60 Hz, CH), 7.84 (1 H, d, ^{3}J = 1.60 Hz, CH), 7.46 (1 H, m, ^{3}J = 10.80 Hz, CH), 6.73 (1 H, d, ^{3}J = 4.40 Hz, CH), 2.65 (3 H, s, Me). LCMS: *m/z* (%) = 240.1 [M+1]. Anal. Calcd for $C_{10}H_8BrNO$ (236.98): C, 50.45; H, 3.39; Br, 33.56; N, 5.88; O, 6.72. Found: C, 50.61; H, 3.44; Br, 33.68; N, 5.97, O, 6.93.

1-Benzyl-5-bromo-1*H*-indole (2d) [3]

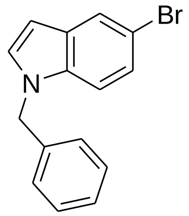

Light Pink solid; yield 0.80g (2.79 mmol, 87%). ^{1}H NMR (300 MHz, $CDCl_3$) δ = 7.79 (1 H, d, ^3J = 1.51 Hz, CH), 7.21-7.37 (4 H, m, 4 CH), 7.09-7.17 (4 H, m, 4 CH), 6.51 (1 H, dd, ^3J = 0.76, 3.02 Hz, CH), 5.32 (2 H, s, CH_2). LCMS: *m/z* (%) = 288.0 [M+1]. Anal. Calcd for $C_{15}H_{12}BrN$ (285.02): C, 62.96; H, 4.23; Br, 27.92; N, 4.89. Found: C, 63.10; H, 4.29; Br, 27.94; N, 4.95.

5-Bromo-1-methyl-1*H*-indazole (2e)

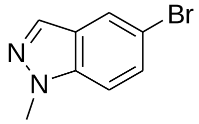

Off white solid; yield 0.15g (0.71 mmol, 77%) ^{1}H NMR (400 MHz, $CDCl_3$) δ = 7.95 (1 H, d, ^3J = 1.00 Hz, CH), 7.66-7.68 (1 H, m, CH), 7.56 (1 H, dd, ^3J = 1.50, 8.76 Hz, CH), 7.36 (1 H, d, ^3J = 8.76 Hz, CH), 4.01 (3 H, s, Me). LCMS: *m/z* (%) = 212.2 [M+1]. Anal. Calcd for $C_8H_7BrN_2$ (209.98): C, 45.53; H, 3.34; Br, 37.86; N, 13.27. Found: C, 45.75; H, 3.53; Br, 37.89; N, 13.31.

5-Bromo-1-isobutyl-1*H*-indazole (2f)

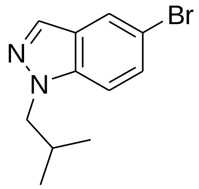

Off white solid; yield 0.11g (0.43 mmol, 70%). ^{1}H NMR (300 MHz, $CDCl_3$) δ = 8.55 (1 H, d, ^3J = 1.75 Hz, CH), 7.67 (1 H, s, CH), 7.11 (1 H, dd, ^3J = 3.22, 4.82 Hz, CH), 6.92 (1 H, dd, ^3J = 11.76, 17.66 Hz, CH), 3.83-3.91 (2 H, s, CH_2), 2.08-2.32 (1 H, m, CH), 0.94 (6 H, dd, ^3J = 3.26, 6.66 Hz, 2 Me). LCMS: *m/z* = 253.14 [M+1]. Anal. Calcd for $C_{11}H_{13}BrN_2$ (252.03): C, 52.19; H, 5.18; Br, 31.57; N, 11.07. Found: C, 52.23; H, 5.35; Br, 31.82; N, 11.30.

1-(5-Bromo-1*H*-indazol-1-yl)ethanone (2g) [6]

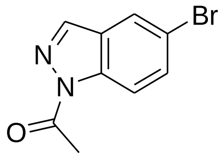

Off white solid; yield 0.18g (0.75 mmol, 58%). ^{1}H NMR (400 MHz, DMSO-d6): δ = 8.42 (1 H, d, ^3J = 8.80 Hz, CH), 7.65 (1 H, d, ^3J = 1.60 Hz, CH), 7.45 (1 H, m, ^3J = 10.80 Hz, CH), 6.75 (1 H, d, ^3J = 4.40 Hz, CH), 2.65 (3 H, s, Me). LCMS: *m/z* (%) = 241.0 [M+1]. Anal. Calcd for $C_{10}H_8BrNO$ (237.97): C, 45.22; H, 2.95; Br, 33.42; N, 11.72; O, 6.69. Found: C, 45.52; H, 3.03; Br, 33.51; N, 11.99, O, 6.70.

1-Benzyl-5-bromo-1*H*-indazole (2h) [4]

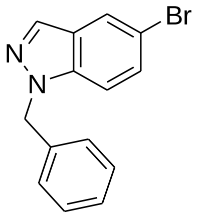

Colorless viscous liquid; yield 1.2g (4.17 mmol, 92%). ^{1}H NMR (300 MHz, $CDCl_3$) δ = 8.00 (1 H, d, *J* = 0.76 Hz, CH), 7.80-7.93 (1 H, m, CH), 7.37-7.47 (1 H, m, CH), 7.26-7.34 (3 H, m, 3 CH), 7.13-7.23 (3 H, m, 3 CH), 5.60 (2 H, s, CH2). LCMS: *m/z* (%) = 288.2 [M+1]. Anal. Calcd for $C_{14}H_{11}BrN_2$ (286.01): C, 58.56; H, 3.86; Br, 27.83; N, 9.76. Found: C, 58.70; H, 3.88; Br, 27.87; N, 9.77.

5-Bromo-2-methyl-2*H*-indazole (2i)

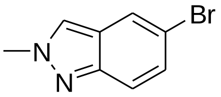

Off white solid; yield 0.10g (0.47 mmol, 69%) ^{1}H NMR (400 MHz, $CDCl_3$) δ = 7.95 (1 H, d, ^3J = 1.00 Hz, CH), 7.66-7.68 (1 H, m, CH), 7.56 (1 H, dd, ^3J = 1.50, 8.76 Hz, CH), 7.33 (1 H, d, ^3J = 8.76 Hz, CH), 4.05 (3 H, s, Me). LCMS: *m/z* (%) = 212.2 [M+1]. Anal. Calcd for $C_8H_7BrN_2$ (211.06): C, 45.53; H, 3.34; Br, 37.86; N, 13.27. Found: C, 45.62; H, 3.45; Br, 37.80; N, 13.33.

5-Bromo-2-isobutyl-2*H*-indazole (2j)

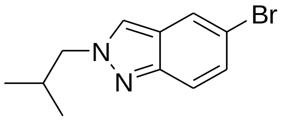

Colorless viscous liquid; yield 0.180g (0.71 mmol, 89%). ^{1}H NMR (300 MHz, $CDCl_3$) δ = 8.54 (1 H, d, ^3J = 1.75 Hz, CH), 7.60 (1 H, s, CH), 7.12 (1 H, dd, ^3J = 3.22, 4.82 Hz, CH), 6.92 (1 H, dd, ^3J = 11.76, 17.66 Hz, CH), 3.84-3.93 (2 H, s, CH_2), 2.10-2.34 (1 H, m, CH), 0.92 (6 H, dd, ^3J = 3.26, 6.66 Hz, 2 Me). LCMS: m/z 254.2 [M+1]. Anal. Calcd for $C_{11}H_{13}BrN_2$ (252.03): C, 52.19; H, 5.18; Br, 31.57; N, 11.07. Found: C, 52.31; H, 5.30; Br, 31.63; N, 11.09.

2-Benzyl-5-bromo-2*H*-indazole (2k) [4]

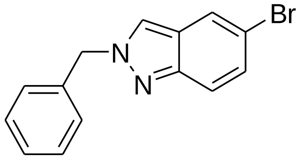

Off white solid; yield 0.75g (2.6 mmol, 88%). ^{1}H NMR (300 MHz, CDCl3) δ = 7.85 (1 H, s, CH), 7.80 (1 H, d, ^3J = 1.13 Hz, CH), 7.63 (1 H, d, ^3J = 9.07 Hz, CH), 7.23-7.40 (6 H, m, 6 CH), 5.60 (2 H, s, CH2). LCMS: *m/z* (%) = 289.02 [M+1]. Anal. Calcd for $C_{14}H_{11}BrN_2$ (286.01): C, 58.56; H, 3.86; Br, 27.83; N, 9.76. Found: C, 58.81; H, 3.97; Br, 27.87; N, 9.83.

Analytical data for compounds 3a–k

General procedure for the synthesis of N-alkylated vinylindole/indazole derivatives (3)[7-9]

To a solution of *N*-alkyl bromoindole/indazoles (**2**, 2.4 mmol, 1 equiv) in DMF (5 mL) under a nitrogen atmosphere was added CS_2CO_3 (7.2 mmol, 3 equiv), potassium vinyltrifluoroborate (4.8 mmol, 2 equiv) and $PdCl_2(dppf)CH_2Cl_2$ adduct (0.24 mmol, 0.1 equiv). The reaction mass was heated to 90 °C for 18 h. When the reaction was completed [TLC (EtOAc/hexane 2:5)], the mixture was extracted with EtOAc (2 × 20 mL) and washed with water (2 × 20 mL). The organic layer was dried over anhydrous Na_2SO_4, filtered and concentrated under reduced pressure. The residue was purified by flash column chromatography [silica gel (230–400 mesh; Merck), EtOAc/hexane 2:5].

1-Methyl-5-vinyl-1*H*-indole (3a) [7]

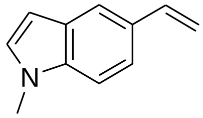

Off white solid; yield 0.21g (1.33 mmol, 75%) ^{1}H NMR (300 MHz, $CDCl_3$) δ = 7.63 (1 H, s, CH), 7.33-7.40 (1 H, m, CH), 7.28 (1 H, s, CH), 7.03 (1 H, d, ^3J = 3.12 Hz, CH), 6.84 (1 H, dd, ^3J = 10.95, 17.56 Hz, CH), 6.46 (1 H, d, ^3J = 2.93 Hz, CH), 5.69 (1 H, d, ^3J = 17.56 Hz, CH), 5.13 (1 H, ^3J = 10.76 Hz, CH), 3.79 (3 H, s, Me). LCMS: *m/z* (%) = 158.08 [M+1]. Anal. Calcd for $C_{11}H_{11}N$ (157.09): C, 84.04; H, 7.05; N, 8.91. Found: C, 84.54; H, 7.35; N, 8.99.

1-Isobutyl-5-vinyl-1*H*-indole (3b)

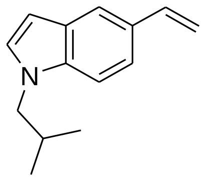

Off white solid; yield 0.19g (0.95 mmol, 73%). ^{1}H NMR (300 MHz, $CDCl_3$) δ = 7.74 (1 H, d, ^3J = 1.70 Hz, CH), 7.62 (1 H, s, CH), 7.30-7.36 (1 H, m, CH), 7.22-7.29 (1 H, m, CH), 7.05 (1 H, dd, ^3J = 3.12, 4.72 Hz, CH), 6.83 (1 H, dd, ^3J = 10.86, 17.56 Hz, CH), 6.44 (1 H, dd, ^3J = 3.12, 13.12 Hz, CH), 5.68 (1 H, dd, ^3J = 0.85, 17.56 Hz, CH), 5.12 (1 H, dd, ^3J = 0.90, 10.91 Hz, CH), 3.83-3.91 (2 H, s, CH_2), 2.11-2.25 (1 H, m, CH), 0.91 (6 H, dd, ^3J = 3.26, 6.66 Hz, 2 Me). LCMS: *m/z* (%) = 200.14 [M+1]. Anal. Calcd for $C_{14}H_{17}N$ (199.14): C, 85.37; H, 9.60; N, 7.03. Found: C, 85.52; H, 9.75; N, 7.33

1-(5-Vinyl-1*H*-indol-1-yl)ethanone (3c) [8]

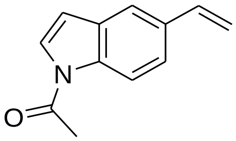

Off white solid; yield 0.21g (1.13 mmol, 78%). ^{1}H NMR (300 MHz, $CDCl_3$) δ = 8.37 (1 H, d, ^3J = 8.69 Hz, CH), 7.58 (1 H, s, CH), 7.38-7.49 (2 H, m, 2 CH), 6.81 (1 H, dd, ^3J = 10.91, 17.61 Hz, CH), 6.63 (1 H, d, ^3J = 3.78 Hz, CH), 5.77 (1 H, d, ^3J = 17.56 Hz, CH), 5.24 (1 H, d, ^3J = 10.86 Hz, CH), 2.64 (3 H, s, Me), 1.54 (3 H, s, Me). LCMS: *m/z* (%) = 186.2 [M+1]. Anal. Calcd for $C_{12}H_{11}NO$ (185.08): C, 77.81; H, 5.99; N, 7.56; O, 8.64. Found: C, 77.82; H, 6.10; N, 7.56; O, 8.73.

1-Benzyl-5-vinyl-1*H*-indole (3d) [9]

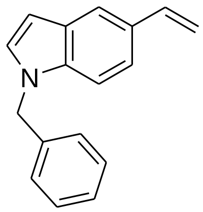

Off white solid; yield 0.52g (2.23 mmol, 81%). ^{1}H NMR (400 MHz, $CDCl_3$) δ = 7.65 (1 H, d, ^3J = 1.75 Hz, CH), 7.24-7.33 (3 H, m, 3 CH), 7.19-7.23 (2 H, m, 2 CH), 7.07-7.14 (3 H, m, CH), 6.83 (1 H, dd, ^3J = 11.13, 17.39 Hz, CH), 6.53 (1 H, dd, ^3J = 0.88, 3.13 Hz, CH), 5.68 (1 H, dd, ^3J = 1.00, 17.51 Hz, CH), 5.58 (2 H, s, CH_2), 5.08-5.16 (1 H, m, CH). LCMS: *m/z* (%) = 234.2 [M+1]. Anal. Calcd for $C_{17}H_{15}N$ (233.12): C, 87.52; H, 6.48; N, 6.00. Found: C, 87.59; H, 6.50; N, 6.23.

1-Methyl-5-vinyl-1*H*-indazole (3e)

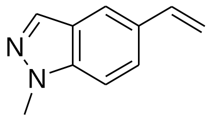

Off white solid; yield 0.18g (1.13 mmol, 78%) ^{1}H NMR (400 MHz, $CDCl_3$) δ = 7.95 (1 H, d, ^3J = 1.00 Hz, CH), 7.66-7.68 (1 H, m, CH), 7.55 (1 H, dd, ^3J = 1.50, 8.76 Hz, CH), 7.34 (1 H, d, ^3J = 8.76 Hz, CH), 6.82 (1 H, dd, ^3J = 10.76, 17.51 Hz, CH), 5.73 (1 H, dd, ^3J = 0.88, 17.64 Hz, CH), 5.21 (1 H, dd, ^3J = 0.75, 10.76 Hz, CH), 4.06 (3 H, s, Me). LCMS: *m/z* (%) = 159.2 [M+1]. Anal. Calcd for $C_{10}H_{10}N_2$ (158.08): C, 75.92; H, 6.37; N, 17.71. Found: C, 75.88; H, 6.83; N, 17.58.

1-Isobutyl-5-vinyl-1*H*-indazole (3f)

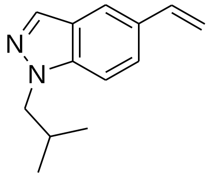

Off white solid; yield 0.18g (0.89 mmol, 65%). ^{1}H NMR (300 MHz, $CDCl_3$) δ = 8.54 (1 H, d, ^3J = 1.75 Hz, CH), 7.62 (1 H, s, CH), 7.10 (1 H, dd, ^3J = 3.22, 4.82 Hz, CH), 6.92 (1 H, dd, ^3J = 11.76, 17.66 Hz, CH), 6.52 (1 H, dd, ^3J = 3.12, 13.12 Hz, CH), 5.76 (1 H, dd, ^3J = 0.85, 17.56 Hz, CH), 5.20 (1 H, dd, ^3J = 0.90, 10.91 Hz, CH), 3.83-3.91 (2 H, s, CH_2), 2.09-2.33 (1 H, m,

CH), 0.92 (6 H, dd, ^3J = 3.26, 6.66 Hz, 2 Me). LCMS: *m/z* (%) = 201.2 [M+1]. Anal. Calcd for $C_{13}H_{16}N_2$ (200.13): C, 77.96; H, 8.05; N, 13.99. Found: C, 78.00; H, 8.25; N, 14.09.

1-(5-Vinyl-1*H*-indazol-1-yl)ethanone (3g)

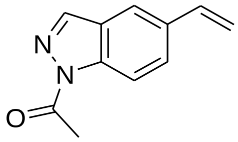

Pale yellow solid; yield 0.20g (1.07 mmol, 80%). ^{1}H NMR (300 MHz, $CDCl_3$) δ = 8.42 (1 H, d, ^3J = 8.69 Hz, CH), 7.42-7.51 (2 H, m, CH), 6.81 (1 H, dd, ^3J = 10.91, 17.61 Hz, CH), 6.70 (1 H, d, ^3J = 3.78 Hz, CH), 5.87 (1 H, d, ^3J = 17.56 Hz, CH), 5.32 (1 H, d, ^3J = 10.86 Hz, CH), 2.68 (3 H, s, Me), 1.62 (3 H, s, Me). LCMS: *m/z* (%) = 187.2 [M+1]. Anal. Calcd for $C_{11}H_{10}N_2O$ (186.08): C, 70.95; H, 5.41; N, 15.04; O, 8.59. Found: C, 71.02; H, 5.62; N, 15.53; O, 8.71.

1-Benzyl-5-vinyl-1*H*-indazole (3h)

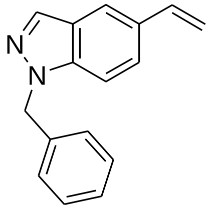

Pale yellow solid; yield 0.46g (1.96 mmol, 82%). ^{1}H NMR (400 MHz, $CDCl_3$) δ = 8.01 (1 H, d, ^3J = 0.75 Hz, CH), 7.65-7.71 (1 H, m, CH), 7.49 (1 H, dd, ^3J = 1.50, 8.76 Hz, CH), 7.26-7.32 (4 H, m, CH), 7.16-7.20 (1 H, m, CH) 6.81 (1 H, dd, ^3J = 11.6, 17.51 Hz, CH), 5.70 (1 H, dd, ^3J = 0.75, 17.51 Hz, CH), 5.58 (2 H, s, CH_2), 5.20 (1 H, dd, ^3J = 0.75, 11.01 Hz, CH). LCMS: *m/z* (%) = 235.2 [M+1]. Anal. Calcd for $C_{16}H_{14}N_2$ (234.12): C, 82.02; H, 6.02; N, 11.96. Found: C, 82.32; H, 6.47; N, 12.06.

2-Methyl-5-vinyl-2*H*-indazole (3i)

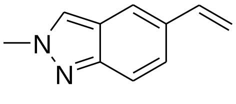

Off white solid; yield 0.13g (0.82 mmol, 73%). ^{1}H NMR (400 MHz, $CDCl_3$) δ = 7.86 (1 H, s, CH), 7.64 (1 H, d, ^3J = 9.07 Hz, CH), 7.45-7.56 (2 H, m, 2 CH), 6.79 (1 H, dd, ^3J = 10.95, 17.37 Hz, CH), 5.71 (1 H, d, ^3J = 17.37 Hz, CH), 5.20 (1 H, d, ^3J = 10.95 Hz, CH), 4.20 (3 H, s, Me).

LCMS: *m/z* (%) = 159.2 [M+1]. Anal. Calcd for $C_{10}H_{10}N_2$ (158.08): C, 75.92; H, 6.37; N, 17.71. Found: C, 76.00; H, 6.50; N, 17.92.

2-Isobutyl-5-vinyl-2*H*-indazole (3j)

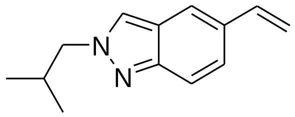

Pale yellow solid; yield 0.13g (0.64 mmol, 75%). ^{1}H NMR (300 MHz, $CDCl_3$) δ = 8.54 (1 H, d, ^3J = 1.75 Hz, CH), 7.60 (1 H, s, CH), 7.12 (1 H, dd, ^3J = 3.22, 4.82 Hz, CH), 6.92 (1 H, dd, ^3J = 11.76, 17.66 Hz, CH), 6.53 (1 H, dd, ^3J = 3.12, 13.12 Hz, CH), 5.76 (1 H, dd, ^3J = 0.85, 17.56 Hz, CH), 5.20 (1 H, dd, ^3J = 0.90, 10.91 Hz, CH), 3.84-3.93 (2 H, s, CH_2), 2.10-2.34 (1 H, m, CH), 0.92 (6 H, dd, ^3J = 3.26, 6.66 Hz, 2 Me). LCMS: *m/z* = 201.2 [M+1]. Anal. Calcd for $C_{13}H_{16}N_2$ (200.13): C, 77.96; H, 8.05; N, 13.99. Found: C, 78.20; H, 8.27; N, 13.98.

2-Benzyl-5-vinyl-2*H*-indazole (3k)

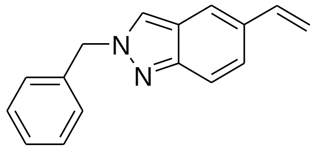

Pale yellow solid; yield 0.320g (1.36 mmol, 72%). ^{1}H NMR (400 MHz, $CDCl_3$) δ = 7.95 (1 H, d, ^3J = 0.75 Hz, CH), 7.68-7.73 (1 H, m, CH), 7.53 (2 H, dd, ^3J = 1.50, 8.76 Hz, 2 CH), 7.34-7.40 (5 H, m, CH), 6.84 (1 H, dd, ^3J = 11.6, 17.51 Hz, CH), 5.73 (1 H, dd, ^3J = 0.75, 17.51 Hz, CH), 5.67 (2 H, s, CH_2), 5.21 (1 H, dd, ^3J = 0.75, 11.01 Hz, CH). LCMS: *m/z* = 235.2 [M+1]. Anal. Calcd for $C_{16}H_{14}N_2$ (234.12): C, 82.02; H, 6.02; N, 11.96. Found: C, 82.12; H, 6.20; N, 12.22.

Analytical data for compounds 7a–k and 8a–k

General procedure for the preparation of spiropyrrolidine derivatives compounds (7 and 8).

To the solution of **3** (1 mmol, 1 equiv) in dry methanol (5 mL) in a single-necked round-bottomed flask, ninhydrin (1.2 mmol, 1.2 equiv) and L-proline/sarcosine (1.2 mmol, 1.2 equiv) were added. The reaction mixture was heated to 60 °C for 30 min and reaction progress was monitored by TLC (EtOAc/hexanes 1:1). After completion of the reaction, the organic solvent was evaporated to dryness. Water (10 mL) was added to the residue and extracted twice with

ethyl acetate (2 × 10 mL) and solvent was removed under vacuum. The crude product obtained was purified by flash chromatography on silica gel (EtOAc/hexane 1:1) to get corresponding spiropyrrolidine derivatives **7** and **8**.

1'-Methyl-3'-(1-methyl-1*H*-indol-5-yl)spiro[indene-2,2'-pyrrolidine]-1,3-dione (7a)

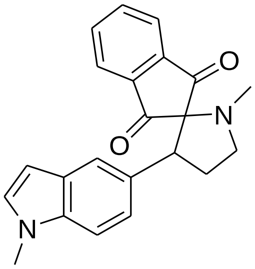

Yellow solid; yield 0.07g (0.20 mmol, 90%). ^{1}H NMR(400 MHz, CDCl3): δ = 7.83 (1 H, d, ^3J = 10.00 Hz, CH), 7.51-7.64 (3 H, m, 3 CH), 7.28 (1 H, d, ^3J = 14.40 Hz, CH), 6.86-6.97 (3 H, m, 3 CH), 6.27 (1 H, d, ^3J = 4.00 Hz, CH), 3.99 (1 H, q, ^3J = 26.00 Hz, CH), 3.63 (3 H, s, 3 CH), 3.55 (1 H, q, ^3J = 19.60 Hz, CH), 3.38-3.46 (1 H, m, CH), 2.81-2.87 (1 H, m, CH), 2.40-2.46 (1 H, m, CH), 2.36 (3 H, s, Me). ^{13}C NMR (400 MHz): δ = 20.40, 32.22, 38.00, 42.11, 52.42, 102.71, 104.26, 111.21, 117.60, 119.33, 128.59, 128.92, 128.72, 128.77, 133.15, 133.21, 141.29, 141.33, 197.45, 197.74. LCMS: *m/z* (%) = 345.1 [M+1]. Anal. Calcd for $C_{22}H_{20}N_2O_2$ (344.15): C, 76.72; H, 5.85; N, 8.13; O, 9.29. Found: C, 76.80; H, 5.89; N, 8.30; O, 9.41.

3'-(1-Isobutyl-1*H*-indol-5-yl)-1'-methylspiro[indene-2,2'-pyrrolidine]-1,3-dione (7b)

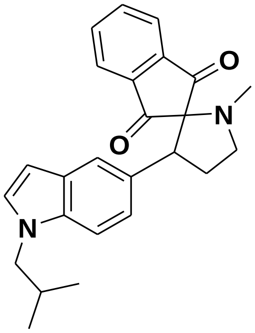

Yellow solid; yield 0.058g (0.15 mmol, 93%). ^{1}H-NMR(400 MHz, MeOD): δ = 7.82 (1 H, q, ^3J = 7.60 Hz, CH), 7.68-7.72 (1 H, m, CH), 7.61 (1 H, t, ^3J = 16.00 Hz, CH), 7.45-7.47 (1 H, m, CH), 7.20 (1 H, d, ^3J = 1.60 Hz, CH), 6.99-7.05 (2 H, m, 2 CH), 6.73-6.76 (1 H, m, CH), 6.24 (1 H, q, ^3J = 3.60 Hz, CH), 3.90 (1 H, q, ^3J = 11.20 Hz, CH), 3.80 (2 H, d, ^3J = 7.60 Hz, CH_2), 3.49-3.51 (2 H, m, CH_2), 2.82-2.90 (1 H, m, CH), 2.41-2.47 (1 H, m, CH), 2.33 (3 H, s, Me), 2.02-2.08 (1

H, m, CH), 0.77-0.80 (6 H, m, 2 Me), ^{13}C NMR (400 MHz): δ = 20.36, 30.66, 30.88, 36.92, 54.52, 54.98, 57.47, 81.90, 101.51, 110.36, 121.11, 122.58, 123.05, 123.39, 127.18, 129.77, 130.02, 136.85, 137.21, 137.29, 142.73, 143.19, 203.74, 204.49. LCMS: *m/z* (%) = 387.2 [M+1]. Anal. Calcd for $C_{25}H_{26}N_2O_2$ (386.2): C, 77.69; H, 6.78; N, 7.25; O, 8.28. Found: C, 77.76; H, 6.80; N, 7.32; O, 8.40.

3'-(1-Acetyl-1*H*-indol-5-yl)-1'-methylspiro[indene-2,2'-pyrrolidine]-1,3-dione (7c)

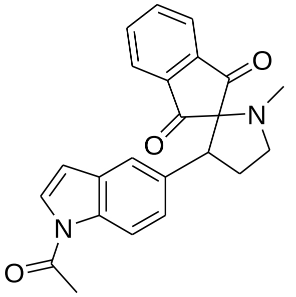

Pale yellow solid; yield 0.076g (0.20 mmol, 87%). 1H NMR (400 MHz, MeOD) δ = 7.98-8.03 (1 H, m, CH), 7.85-7.88 (1 H, m, CH), 7.73-7.79 (1 H, m, CH), 7.68 (1 H, dt, ^3J = 1.26, 7.40 Hz CH), 7.50-7.58 (2 H, m, 2 CH), 7.26 (1 H, d, ^3J=2.01 Hz, CH), 6.93 (1 H, dd, ^3J = 2.01, 8.53 Hz, CH), 6.49-6.53 (1 H, m, CH), 3.92 (1 H, dd, ^3J=8.28, 11.29 Hz, CH), 3.45-53 (1 H, m, CH), 3.36.3.45 (1 H, m, CH), 2.78-2.90 (1 H, m, CH), 2.56(3 H, s, Me), 2.44 (1 H, dtd, ^3J = 4.02, 8.16, 12.30 Hz, CH), 2.32 (3 H, s, Me), ^{13}C NMR (400 MHz): δ = 23.648, 23.828, 30.653, 30.852, 36.839, 54.995, 55.136, 56.652, 82.342, 109.600, 109.820, 117.264, 121.426, 123.173, 123.575, 126.113, 127.778, 132.363, 133.452, 137.244, 137.652, 142.423, 142.673, LCMS: *m/z* (%) = 374 [M+1]. Anal. Calcd for $C_{23}H_{20}N_2O_3$ (372.15): C, 74.18; H, 5.41; N, 7.52; O, 12.89. Found: C, 74.26; H, 5.49; N, 7.55; O, 12.85.

3’-(1-Benzyl-1*H*-indol-5-yl)-1’-methylspiro[indene-2,2’-pyrrolidine]-1,3-dione (7d)

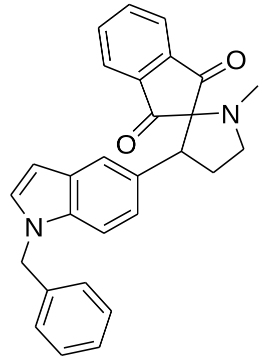

Pale yellow solid; yield 0.093g (0.22 mmol, 91%). ^{1}H NMR(300 MHz, DMSO): δ = 7.66-7.86 (3 H, m, 3 CH), 7.50 (1 H, d, ^{3}J = 7.55 Hz, CH), 7.32 (1 H, d, ^{3}J = 3.02 Hz, CH), 7.14-7.28 (4 H, m, 4 CH), 7.08 (1 H, d, ^{3}J = 8.69 Hz, CH), 6.97 (2 H, d, ^{3}J = 6.04 Hz, CH), 6.64 (1 H, d, ^{3}J = 8.69 Hz, CH), 6.30 (1 H, d, ^{3}J = 3.02 Hz, CH), 5.25 (2 H, s, CH_2), 3.80 (1 H, dd, ^{3}J = 8.31, 11.33 Hz, CH), 3.35-3.41 (1 H, m, CH), 3.19-3.28 (1 H, m, CH), 2.62-2.76 (1 H, m, CH), 2.25-2.36 (1 H, m, CH), 2.14-2.21 (3 H, m, CH_3). ^{13}C NMR (300 MHz): δ = 30.0, 36.7, 49.9, 54.0, 56.0, 76.5, 77.2, 77.4, 80.6, 101.6, 109.2, 120.5, 122.0, 122.2, 122.4, 126.5, 127.0, 127.5, 128.3, 128.4, 128.6, 135.0, 135.5, 135.5, 137.4, 141.3, 141.7, 203.1, 203.8. LCMS: *m/z* (%) = 421.2 [M+1]. Anal. Calcd for $C_{28}H_{24}N_2O_2$ (420.18): C, 79.98; H, 5.75; N, 6.66; O, 7.61. Found: C, 80.12; H, 5.95; N, 6.76; O, 7.65.

1’-Methyl-3’-(1-methyl-1*H*-indazol-5-yl)spiro[indene-2,2’-pyrrolidine]-1,3-dione (7e)

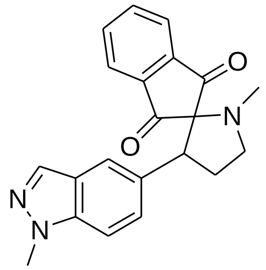

Pale yellow solid; yield 0.068g (0.19 mmol, 85%). ^{1}H NMR (400 MHz, MeOD) δ = 7.85-7.89 (1 H, m, CH), 7.82 (1 H, d, ^{3}J = I.00 Hz, CH), 7.73-7 79 (1H, m, CH), 7.65-7.71 (1 H, m, CH), 7.52 (1 H, td, ^{3}J = 1.00, 7.53 Hz, CH), 7.44 (1 H, d, ^{3}J = 1.00 Hz, CH), 7.19-7.24 (1 H, m, CH), 7.06 (1 H, dd, ^{3}J = 1.51, 9.04 Hz, CH), 3.92-3.98 (1 H, m, CH), 3.91 (3 H, s, Me), 3.46-3.53 (1 H, m, CH), 3.37-3.45 (1 H, m, CH), 2.84 (1 H, dddd, ^{3}J = 7.03, 9.91, 11.23, 12.49 Hz, CH), 2.45 (1 H, dtd, ^{3}J = 3.76, 8.09, 12.17 Hz, CH), 2.32 (3 H, s, Me), ^{13}C NMR (400 MHz): δ = 30.384, 36.819,

40.137, 54.955, 56.840, 81.180, 117.097, 120.691, 122.952, 123.156, 123.571, 126.214, 128.289, 130.404, 137.234, 137.644, 142.623, 143.035, 149.241, 203.465, 204.125. LCMS: *m/z* (%) = 347 [M+1]. Anal. Calcd for $C_{21}H_{19}N_3O_2$ (345.15): C, 73.03; H, 5.54; N, 12.17; O, 9.26. Found: C, 73.20; H, 5.63; N, 12.15; O, 9.27.

3'-(1-Isobutyl-1*H*-indazol-5-yl)-1'-methylspiro[indene-2,2'-pyrrolidine]-1,3-dione (7f)

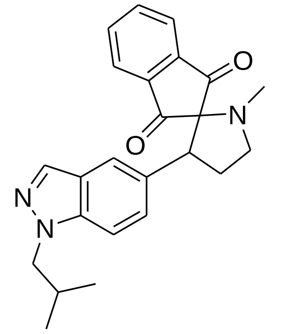

Pale yellow solid; yield 0.070g (0.18 mmol, 82%). ^{1}H NMR (300 MHz, MeOD) δ = 7.82-7.87 (2 H, m, 2 CH), 7.73 (1 H, dt, ^3J =1.00, 7.53 Hz, CH), 7.64 (1 H, dt, ^3J = 1.00, 7.53 Hz, CH), 7.42-7.49 (2 H, m, 2 CH), 7.21 (1 H, d, ^3J = 9.04 Hz, CH), 7.03 (1 H, dd, ^3J = 1.51, 9.04 Hz, CH), 4.06 (2 H, d, ^3J = 7.53 Hz, CH_2), 3.94 (1 H, dd, ^3J = 8.28, 11.29 Hz, CH), 3.47-3.54 (1 H, m, CH), 3.39 (1 H, m, CH), 2.79-2.91 (1 H, m, CH), 2.45 (1 H, dtd, ^3J = 3.76, 8.03, 12.30 Hz, CH), 2.33 (3 H, s, Me), 2.15 (1 H, td, ^3J = 6.96, 13.68 Hz, CH), 0.78 (6 H, dd, ^3J = 6.78, 8.28 Hz, 2 Me). ^{13}C NMR (400 MHz): δ = 20.46, 20.66, 20.68, 26.52, 31.82, 38.01, 52.40, 60.11, 104.21, 113.62, 120.23, 124.58, 125.65, 128.70, 130.45, 133.01, 133.12, 133.16, 137.00, 141.20, 141.25, 197.63, 197.65. LCMS: *m/z* (%) = 389 [M+1]. Anal. Calcd for $C_{24}H_{25}N_3O_2$ (387.19): C, 74.39; H, 6.50; N, 10.84; O, 8.26. Found: C, 74.43; H, 6.59; N, 10.87; O, 8.30.

3’-(1-Acetyl-1*H*-indazol-5-yl)-1’-methylspiro[indene-2,2’-pyrrolidine]-1,3-dione (7g)

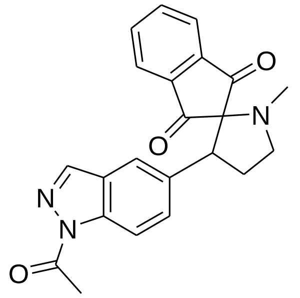

Pale yellow solid; yield 0.069g (0.18 mmol, 78%). ^{1}H NMR(300 MHz, MeOD): δ = 8.11 (1 H, d, ^3J = 0.90 Hz, CH), 8.02 (1 H, d, ^3J = 8.70 Hz, CH), 7.89 (1 H, t, ^3J = 7.20 Hz, CH), 7.68-7.82 (2 H, m, 2 CH), 7.54 (2 H, t, ^3J = 7.20 Hz, 2 CH), 7.22 (1 H, dd, ^3J = 10.50, Hz, CH), 3.98 (1 H, q, ^3J = 19.50 Hz, CH), 3.43-3.53 (2 H, m, CH_2), 2.82-2.85 (1 H, m, CH), 2.67 (3 H, s, Me), 2.46-2.51 (1 H, m, CH), 2.33 (3 H, s, Me), ^{13}C NMR (300 MHz): δ = 22.74, 30.68, 36.79, 55.00, 56.15, 81.00, 115.79, 121.75, 123.21, 123.69, 127.82, 130.99, 134.27, 137.45, 137.81, 139.37, 140.93, 142.55, 142.91, 172.38, 203.29, 203.98, LCMS: *m/z* (%) = 374.2 [M+1]. Anal. Calcd for $C_{22}H_{19}N_3O_3$ (373.14): C, 70.76; H, 5.13; N, 11.25; O, 12.85. Found: C, 70.78; H, 5.22; N, 11.28; O, 12.88.

3’-(1-Benzyl-1*H*-indazol-5-yl)-1’-methylspiro[indene-2,2’-pyrrolidine]-1,3-dione (7h)

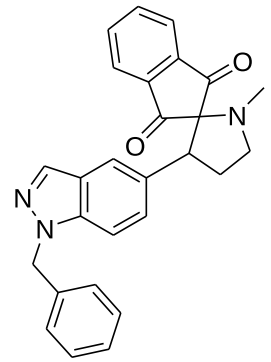

Off white solid; yield 0.073g (0.17 mmol, 79%). ^{1}H NMR (400 MHz, CDCl3) δ = 7.79-7.85 (2 H, m, 2 CH), 7.61 (1 H, dt, ^3J = 1.25, 7.38 Hz, CH), 7.52 (1 H, dt, ^3J = 1.00, 7.38 Hz, CH), 7.39 - 7.47 (2 H, m, 2 CH), 7.20 - 7.25 (3 H, m, 3 CH), 6.97-7.05 (4 H, m, 4 CH), 5.43 (2 H, s, CH_2), 3.96 (1 H, dd, ^3J = 8.13, 11.13 Hz, CH), 3.52-3.60 (1 H, m, CH), 3.43 (1 H, dt, ^3J = 3.63, 9.32 Hz, CH), 2.73-2.86 (1 H, m, CH), 2.39-2.45 (1 H, m, CH), 2.36 (3 H, br. S., CH_3). ^{13}C NMR (400 MHz): δ = 21.0, 31.5, 35.4, 37.8, 52.1, 61.9, 103.8, 114.0, 122.0, 124.6, 126.2, 129.1, 129.3, 130.0, 130.4, 131.7, 133.0, 133.2, 134.5, 134.7, 137.3, 137.9, 142.6, 142.9, 197.6, 197.8. LCMS:

m/z (%) = 422.2 [M+1]. Anal. Calcd for $C_{27}H_{23}N_3O_2$ (421.18): C, 76.94; H, 5.50; N, 9.97; O, 7.59. Found: C, 77.00; H, 5.53; N, 10.09; O, 7.65.

1'-Methyl-3'-(2-methyl-2*H*-indazol-5-yl)spiro[indene-2,2'-pyrrolidine]-1,3-dione (7i)

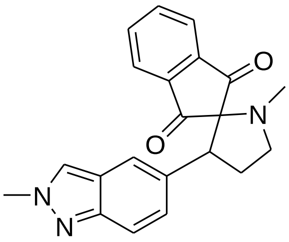

Pale yellow solid; yield 0.048g (0.13 mmol, 70%). ^{1}H NMR (400 MHz, MeOD) δ = 8.01 (1 H, s, CH), 7.89 - 7.85 (1 H, m, CH), 7.79-7.73 (1 H, m, CH), 7.72 - 7.64 (1 H, m, CH), 7.54 - 7.49 (1 H, m, CH), 7.38 (1 H, d, ^3J=1.0 Hz, CH), 7.22 (1 H, d, ^3J=9.0 Hz, CH), 6.91 (1 H, dd, ^3J=9.0, 1.5 Hz, CH), 4.10 (3 H, s, Me), 3.90 (1 H, dd, ^3J= 11.0, 8.0 Hz, CH), 3.54- 3.46 (1 H, m, CH), 3.45-3.37 (1 H, m, CH), 2.90 - 2.76 (1 H, m, CH), 2.42 (1 H, dtd, ^3J=12.1, 8.1, 3.8 Hz, CH), 2.32 (3 H, s, Me), ^{13}C NMR (400 MHz): δ = 30.755, 35.417, 36.838, 54.978, 56.509, 81.127, 110.041, 121.465, 123.157, 123.585, 124.913, 128.433, 129.870, 133.540, 137.276, 137.674, 140.586, 142.624, 143.004, 203.504, 204.183. LCMS: *m/z* (%) = 346 [M+1]. Anal. Calcd for $C_{21}H_{19}N_3O_2$ (345.15): C, 73.03; H, 5.54; N, 12.17; O, 9.26. Found: C, 73.28; H, 5.61; N, 12.20; O, 9.27.

3'-(2-Isobutyl-2*H*-indazol-5-yl)-1'-methylspiro[indene-2,2'-pyrrolidine]-1,3-dione (7j)

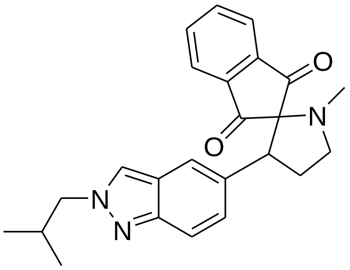

Pale yellow solid; yield 0.050g (0.12 mmol, 65%). ^{1}H NMR (400 MHz, MeOD) δ = 8.03 (1 H, d, ^3J = 0.76 Hz, CH), 7.87 (1 H, td, ^3J = 0.94, 7.55 Hz, CH), 7.75 (1 H, dt, ^3J = 1.32, 7.46 Hz, CH), 7.66 (1 H, dt, ^3J = 1.13, 7.37 Hz, CH), 7.51 (1 H, td, ^3J = 0.94, 7.55 Hz, CH), 7.36-7.41 (1 H, m, CH), 7.23 (1 H, d, ^3J = 9.07 Hz, CH), 6.91 (1 H, dd, ^3J = 1.89, 9.07 Hz, CH), 4.14 (2 H, d, ^3J = 7.18 Hz, CH_2), 3.84-3.95 (1 H, m, CH), 3.36-3.54 (2 H, m, CH_2), 2.75-2.91 (1 H, m, CH), 2.35-2.49 (1 H, m, CH), 2.32 (3 H, s, Me), 2.18-2.29 (1 H, m, CH), 0.87 (6 H, dd, ^3J = 2.46, 6.61 Hz, 2 Me). ^{13}C NMR (400 MHz): δ = 20.46, 20.72, 20.78, 27.12, 31.82, 38.18, 52.47, 58.50, 104.21,

113.63, 120.21, 122.93, 124.50, 125.62, 128.72, 130.42, 133.12, 133.15, 141.20, 141.25, 145.00, 197.63, 197.65. LCMS: *m/z* (%) = 389 [M+1]. Anal. Calcd for $C_{24}H_{25}N_3O_2$, (387.19): C, 74.39; H, 6.50; N, 10.84; O, 8.26. Found: C, 74.40; H, 6.57; N, 10.91; O, 8.30.

3'-(2-Benzyl-2*H*-indazol-5-yl)-1'-methylspiro[indene-2,2'-pyrrolidine]-1,3-dione (7k)

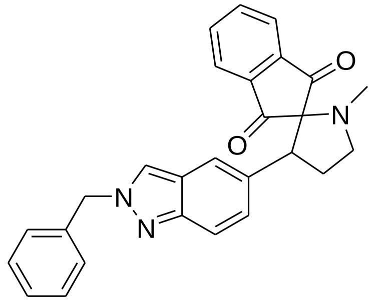

Off white solid; yield 0.048g (0.11 mmol, 60%). ^{1}H NMR(400 MHz, $CDCl_3$) δ = 7.79-7.85 (2 H, m, 2 CH), 7.61 (1 H, dt, ^3J = 1.25, 7.38 Hz, CH), 7.52 (1 H, dt, ^3J = 1.00, 7.38 Hz, CH), 7.39-7.47 (2 H, m, 2 CH), 7.20-7.25 (3 H, m, 3 CH), 6.97-7.05 (4 H, m, 4 CH), 5.43 (2 H, s, CH_2), 3.96 (1 H, dd, ^3J = 8.13, 11.13 Hz, CH), 3.52-3.60 (1 H, m, CH), 3.43 (1 H, dt, ^3J = 3.63, 9.32 Hz CH), 2.73-2.86 (1 H, m, CH), 2.39-2.45 (1 H, m, CH), 2.36 (3 H, br. S., Me). ^{13}C NMR (400 MHz): δ = 22.1, 31.3, 39.2, 54.1, 61.3, 105.3, 112.5, 122.2, 124.8, 126.3, 129.2, 129.4, 130.0, 130.5, 131.7, 133.1, 133.2, 134.6, 134.4, 137.2, 137.1, 142.7, 143.0, 197.7, 197.9. LCMS: *m/z* (%) = 422.2 [M+1]. Anal. Calcd for $C_{27}H_{23}N_3O_2$ (421.18): C, 76.94; H, 5.50; N, 9.97; O, 7.59. Found: C, 77.97; H, 5.53; N, 10.05; O, 7.66.

2'-(1-Methyl-1*H*-indol-5-yl)-1',2',5',6',7',7a'-hexahydrospiro[indene-2,3'-pyrrolizine]-1,3-dione (8a)

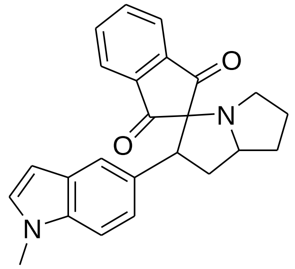

Pale yellow solid; yield 0.062g (0.16 mmol, 86%). ^{1}H NMR(400 MHz, MeOD) δ = 7.82-7.84 (1 H, m, CH), 7.64-7.75 (3 H, m, 3 CH), 7.26 (1 H, s, CH), 7.01-7.26 (2 H, m, 2 CH), 6.92 (1 H, d, ^3J = 63.20 Hz, CH), 6.23 (1 H, q, ^3J = 3.60 Hz, CH), 4.01-0.00 (1 H, m, CH), 3.63 (3 H, d, ^3J =

6.00 Hz, 3 CH), 3.48 (1 H, s, CH), 3.06-3.08 (2 H, m, CH_2), 2.80 (2 H, d, ^3J = 5.20 Hz, CH_2), 2.23-2.23 (1 H, m, CH), 2.10-2.18 (3 H, m, 3 CH), 1.71-1.81 (3 H, m, 3 CH). ^{13}C NMR (400 MHz): δ = 23.4, 29.0, 30.8, 31.9, 42.1, 50.3, 61.7, 99.8, 102.7, 110.9, 117.6, 119.3, 128.0, 128.9, 128.7, 128.7, 131.0, 133.1, 133.1, 133.8, 141.2, 141.2, 197.7, 197.7. LCMS: *m/z* (%) = 373 [M+1]. Anal. Calcd for $C_{24}H_{22}N_2O_2$ (372.18): C, 77.39; H, 6.49; N, 7.52; O, 8.59. Found: C, 77.44; H, 6.55; N, 7.56; O, 8.64.

2'-(1-Isobutyl-1*H*-indol-5-yl)-1',2',5',6',7',7a'-hexahydrospiro[indene-2,3'-pyrrolizine]-1,3-dione (8b)

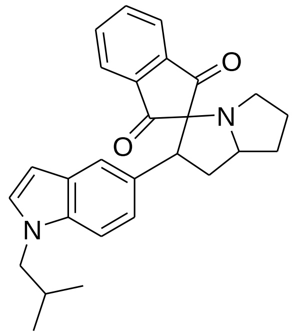

Pale yellow solid; yield 0.070g (0.16 mmol, 80%). ^{1}H NMR(400 MHz, MeOD) δ = 7.79-7.85 (1 H, m, CH), 7.59-7.74 (3 H, m, 3 CH), 7.25 (1 H, d, ^3J=1.51 Hz, CH), 6.99-7.06 (2 H, m, 2 CH), 6.81 (1 H, dd, ^3J = 1.88, 8.66 Hz, CH), 6.23 (1 H, dd, ^3J =0.75, 3.26 Hz, CH), 4.16-4.27 (1 H, m, CH), 3.99 (1 H, dd, ^3J = 7.78, 11.54 Hz, CH), 3.79 (2 H, d, ^3J=7.28 Hz, CH2), 3.38-3.49 (1 H, m, CH), 3.05 (1 H, dt, ^3J = 8.53, 12.17 Hz, 1H), 2.79 (1 H, dt, ^3J=3.26, 6.90 Hz, CH), 1.96-2.30 (4 H, m, 4 CH), 1.62-1.87 (2 H, m, CH2), 0.78 (6 H, dd, ^3J=2.38, 6.65 Hz, 2 CH3) ^{13}C NMR (400 MHz): δ = 21.0, 21.0, 24.4, 29.0, 29.2, 30.0, 31.9, 50.3, 62.2, 66.1, 100.3, 103.8, 110.9, 116.5, 120.3, 128.3, 128.6, 128.6, 128.9, 131.0, 133.1, 133.1, 134.0, 141.9, 141.9, 197.6, 197.6. LCMS: *m/z* (%) = 413.2 [M+1]. Anal. Calcd for $C_{27}H_{28}N_2O_2$ (414.23): C, 78.23; H, 7.29; N, 6.76; O, 7.72. Found: C, 78.31; H, 7.36; N, 6.87; O, 7.73.

2’-(1-Acetyl-1*H*-indol-5-yl)-1’,2’,5’,6’,7’,7a’-hexahydrospiro[indene-2,3’-pyrrolizine]-1,3-dione (8c)

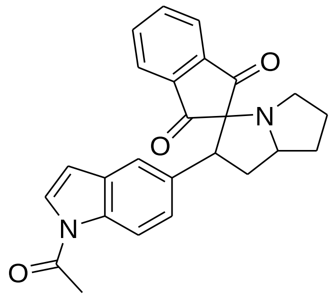

Off white solid; yield 0.081g (0.20 mmol, 83%). ^{1}H NMR (400 MHz, MeOD) δ = 8.05 (1 H, d, ^3J = 8.53 Hz, CH), 7.87-7.90 (1 H, m, CH), 7.72-7.81 (2 H, m, 2 CH), 7.67-7.71 (1 H, m, CH), 7.57 (1 H, d, ^3J = 4.02 Hz, CH), 7.33 (1 H, d, ^3J = 1.51 Hz, CH), 7.02 (1 H, dd, ^3J = 1.76, 8.78 Hz, CH), 6.51-6.55 (1 H, m, CH), 4.22 (1 H, dq, ^3J = 3.01, 8.03 Hz, CH), 4.01 (1 H, dd, ^3J = 7.78, 11.29 Hz, CH), 3.35-3.41 (1 H, m, CH), 3.06 (1 H, ddd, ^3J = 8.53, 11.04, 12.55 Hz, CH), 2.72-2.80 (1 H, m, CH), 2.56 (2 H, s, CH_2), 2.16-2.29 (2 H, m, CH_2), 2.07-2.15 (1 H, m, CH), 1.65-1.86 (2 H, m, CH_2). ^{13}C NMR (400 MHz): δ = 23.4, 24.2, 29.2, 30.5, 31.9, 50.3, 61.7, 99.2, 102.4, 110.9, 117.6, 119.3, 125.5, 127.9, 128.7, 128.7, 131.0, 133.1, 133.1, 133.2, 141.2, 167.8, 197.6. LCMS: *m/z* (%) = 401.2 [M+1]. Anal. Calcd for $C_{25}H_{22}N_2O_3$ (400.18): C, 74.98; H, 6.04; N, 7.00; O, 11.99. Found: C, 75.00; H, 6.10; N, 7.03; O, 12.05.

2’-(1-Benzyl-1*H*-indol-5-yl)-1’,2’,5’,6’,7’,7a’-hexahydrospiro[indene-2,3’-pyrrolizine]-1,3-dione (8d)

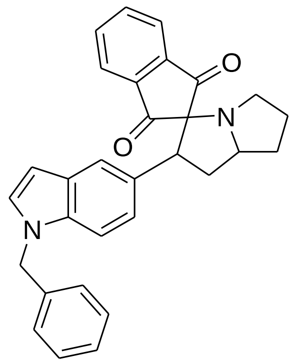

Yellow solid; yield 0.091g (0.20 mmol, 85%). ^{1}H NMR (300 MHz, DMSO-d6) δ = 7.77-7.93 (3 H, m, 3 CH), 7.65-7.74 (1 H, m, CH), 7.35 (1 H, d, ^3J = 3.02 Hz, CH), 7.12-7.29 (6 H, m, 6 CH), 7.07 (2 H, d, ^3J = 6.42 Hz, 2 CH), 6.79 (1 H, d, ^3J = 8.31 Hz, CH), 6.32 (1 H, d, ^3J = 3.02 Hz, CH), 5.26 (2 H, s, CH_2), 3.98 (1 H, br. S., CH), 3.80-3.91 (1 H, m, CH), 2.97-3.08 (1 H, m, CH),

2.84 (1 H, dd, ^3J = 11.90, 19.07 Hz, CH), 1.89-2.16 (3 H, m, 3 CH), 1.44-1.74 (3 H, m, 3 CH). ^{13}C NMR (300 MHz): δ = 27.6, 33.5, 37.1, 49.5, 49.9, 52.4, 65.5, 76.5, 77.0, 77.2, 77.4, 78.3, 101.6, 109.3, 120.7, 122.4, 122.7, 123.2, 126.7, 127.5, 127.6, 128.2, 128.5, 128.6, 135.0, 135.5, 135.5, 137.4, 141.4, 141.6, 200.9. LCMS: *m/z* (%) = 447.2 [M+1]. Anal. Calcd for $C_{25}H_{22}N_2O_3$ (446.54): C, 80.69; H, 5.87; N, 6.27; O, 7.17. Found: C, 80.75; H, 5.89; N, 6.32; O, 7.19.

2’-(1-Methyl-1*H*-indazol-5-yl)-1’,2’,5’,6’,7’,7a’-hexahydrospiro[indene-2,3’-pyrrolizine]-1,3-dione (8e)

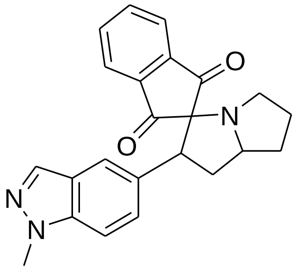

Pale yellow solid; yield 0.077g (0.20 mmol, 86%). ^{1}H NMR (400 MHz, MeOD): δ = 8.02 (1 H, s, CH), 7.88 (1 H, d, ^3J = 9.60 Hz, CH), 7.72-7.80 (2 H, m, 2 CH), 7.67-7.70 (1 H, m, CH), 7.44 (1 H, s, CH), 7.27 (1 H, d, ^3J = 8.80 Hz, CH), 6.99 (1 H, d, ^3J = 2.00 Hz, CH), 4.12 (1 H, d, ^3J = 7.20 Hz, CH), 4.09 (3 H, s, Me), 4.00 (1 H, t, ^3J = 19.20 Hz, CH), 3.40 (1 H, d, ^3J = 6.00 Hz, CH), 3.52 (1 H, q, ^3J = 374.40 Hz, CH), 2.62-2.79 (2 H, m, CH_2), 2.10-2.31 (3 H, m, Me), 1.65-1.89 (2 H, m, CH_2). (300 MHz): δ = 29.8, 34.3, 39.1, 49.9, 50.5, 53.6, 67.2, 76.5, 77.2, 77.2, 77.6, 78.7, 101.8, 110.3, 121.8, 122.5, 122.9, 123.4, 126.9, 128.1, 128.2, 128.7, 128.9, 129.2, 135.0, 135.9, 136.1, 137.8, 141.5, 141.9, 200.9. LCMS: *m/z* (%) = 372.2 [M+1]. Anal. Calcd for $C_{23}H_{21}N_3O_2$ (371.16): C, 74.37; H, 5.70; N, 11.31; O, 8.61. Found: 74.40; H, 5.77; N, 11.44; O, 8.67.

2’-(1-Isobutyl-1*H*-indazol-5-yl)-1’,2’,5’,6’,7’,7a’-hexahydrospiro[indene-2,3’-pyrrolizine]-1,3-dione (8f)

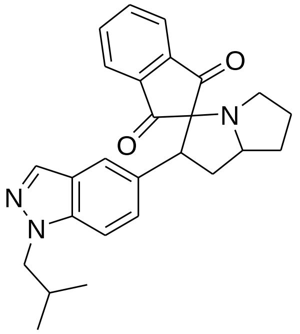

Brown Solid; yield 0.055g (0.13 mmol, 68%). ^{1}H NMR(400 MHz, MeOD): δ = 8.04 (1 H, s, CH), 7.69-8.04 (4 H, m, 4 CH), 7.45 (1 H, s, CH), 7.30 (1H, d, ^3J = 0.03 Hz, CH), 7.15 (1 H, d, ^3J = 12.00 Hz, CH), 7.02 (1 H, d, ^3J = 2.00 Hz, 1H), 4.15 (1 H, d, ^3J = 28.00 Hz, CH), 3.80 (2 H, t, ^3J = 14.00 Hz, CH_2), 3.31-3.32 (1 H, m, CH), 2.62-2.77 (1 H, m, CH), 2.02-2.12 (3 H, m, 3 CH), 1.72-1.89 (2 H, m, 2 CH), 0.87-0.93 (2 H, m, 2 CH), 0.77-0.81 (6 H, m, 2 Me). ^{13}C NMR (400 MHz): δ = 21.0, 21.1, 23.5, 25.5, 29.0, 29.6, 32.0, 51.1, 59.3, 61.2, 99.1, 113.4, 120.2, 125.2, 125.8, 129.0, 129.2, 132.5, 133.2, 133.6, 133.8, 137.3, 141.6, 141.7, 198.2, 198.4. LCMS: *m/z* (%) = 372.2 [M+1]. Anal. Calcd for $C_{26}H_{27}N_3O_2$ (414.2): C, 75.52; H, 6.58; N, 10.16; O, 7.74. Found: 75.60; H, 6.63; N, 10.17; O, 7.82.

2’-(1-Acetyl-1*H*-indazol-5-yl)-1’,2’,5’,6’,7’,7a’-hexahydrospiro[indene-2,3’-pyrrolizine]-1,3-dione (8g)

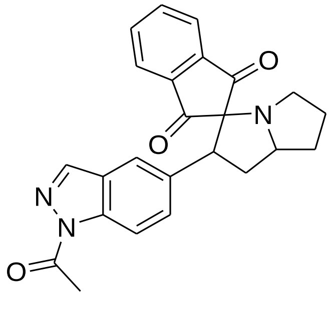

Off white solid; yield 0.090g (0.22 mmol, 70%). ^{1}H NMR(400 MHz, MeOD): δ = 8.06-8.12 (2 H, m, CH), 7.89-7.92 (1 H, m, CH), 7.62-7.82 (3 H, m, 3 CH), 7.62 (1 H, s, CH), 7.30 (1 H, d, ^3J = 2.00 Hz, CH), 4.12 (1 H, t, ^3J = 26.00 Hz, CH), 3.01-3.12 (1 H, m, CH), 2.79 (1 H, t, ^3J = 10.00 Hz, CH), 2.65 (3 H, s, Me), 2.24-2.26 (2 H, m, CH_2), 2.00-2.03 (1 H, m, CH), 1.26-1.31 (2 H, m, CH_2). ^{13}C NMR (400 MHz): δ = 23.5, 23.9, 29.2, 29.6, 32.3, 50.5, 61.9, 99.5, 113.5, 121.1,

124.5, 126.0, 128.0, 128.8, 129.0, 132.4, 133.5, 133.7, 139.1, 143.4, 143.6, 169.0, 197.7, 197.9. LCMS: *m/z* (%) = 400.2 [M+1]. Anal. Calcd for $C_{24}H_{21}N_3O_3$ (399.16): C, 73.00; H, 6.50; N, 11.03; O, 12.52. Found: C, 73.16; H, 6.54; N, 11.12; O, 12.55.

2'-(1-Benzyl-1*H*-indazol-5-yl)-1',2',5',6',7',7a'-hexahydrospiro[indene-2,3'-pyrrolizine]-1,3-dione (8h)

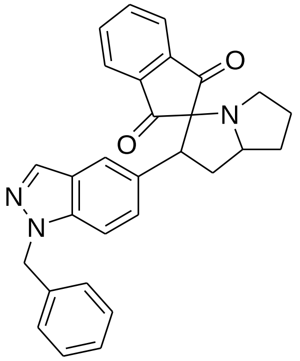

Brown semi solid; yield 0.065g (0.14 mmol, 82%). ^{1}H-NMR(400 MHz, CDCl3): δ = 7.91-7.96 (1 H, m, CH), 7.71-7.82 (4 H, m, 4 CH), 7.42-7.47 (2 H, m, 2 CH), 7.32-7.38 (3 H, m, 3 CH), 7.22-7.26 (2 H, m, 2 CH), 7.02 (1 H, dd, ^3J = 2.01, 9.04 Hz, CH), 5.50 (2 H, s, CH_2), 3.87-3.95 (1 H, m, CH), 3.15-3.24 (1 H, m, CH), 3.01 (1 H, ddd, ^3J = 8.28, 10.42, 12.42 Hz, CH), 2.78-2.86 (1 H, m, CH), 2.05-2.26 (3 H, m, 3 CH), 1.79-1.94 (3 H, m, 3 CH), 1.57-1.72 (1 H, m, CH). ^{13}C NMR (400 MHz): δ = 23.4, 29.0, 29.6, 32.3, 50.3, 61.7, 61.9, 99.3, 114.7, 120.2, 124.3, 126.3, 126.6, 128.6, 128.7, 128.7, 128.9, 131.5, 133.1, 133.5, 133.8, 136.3, 136.5, 141.6, 141.8, 197.2, 197.5. LCMS: *m/z* (%) = 448.2 [M+1]. Anal. Calcd for $C_{29}H_{25}N_3O_2$ (447.19): C, 78.02; H, 6.53; N, 10.00; O, 7.65. Found: C, 78.09; H, 6.63; N, 10.09; O, 7.68.

2'-(2-Methyl-2*H*-indazol-5-yl)-1',2',5',6',7',7a'-hexahydrospiro[indene-2,3'-pyrrolizine]-1,3-dione (8i)

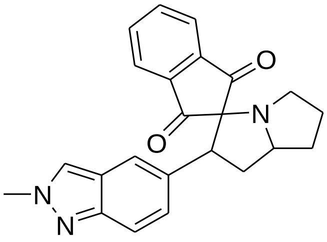

Pale yellow solid; yield 0.072g (0.19 mmol, 68%). ^{1}H NMR(400 MHz, MeOD) δ = 8.02 (1 H, s, CH), 7.86-7.90 (1 H, m, CH), 7.73-7.79 (2 H, m, 2 CH), 7.67-7.70 (1 H, m, CH), 7.43-7.46 (1 H,

m, CH), 7.27 (1 H, d, ^3J = 9.03 Hz, CH), 7.01 (1 H, dd, ^3J = 2.01, 9.04 Hz, CH), 5.50 (1 H, s, CH), 4.13 (1 H, s, CH), 4.11 (3 H, s, Me), 4.00 (1 H, dd, ^3J = 7.78, 11.29 Hz, CH), 3.40 (1 H, dt, ^3J = 6.02, 10.04 Hz, CH), 3.04 (1 H, ddd, ^3J = 8.53, 11.42, 12.67 Hz, CH), 2.79 (1 H, ddd, ^3J = 2.76, 6.90, 9.91 Hz, CH), 2.58-2.76 (1 H, m, CH), 2.23-2.38 (1 H, m, CH), 2.19 (1 H, ddd, ^3J = 3.01, 7.91, 12.67 Hz, CH), 2.08-2.15 (1 H, m, CH), 1.62-1.86 (2 H, m, 2 CH). ^{13}C NMR (125 MHz): δ = 27.4, 29.0, 33.4, 36.2, 40.1, 49.6, 52.0, 58.5, 65.6, 116.9, 119.1, 122.8, 123.2, 123.6, 127.0, 136.0, 141.5, 148.2, 148.2, 200.0, 200.0, 200.2, 203.9. LCMS: *m/z* (%) = 373 [M+1]. Anal. Calcd for $C_{23}H_{21}N_3O_2$ (371.16): C, 75.17; H, 6.00; N, 11.62; O, 8.92. Found: C, 75.22; H, 6.17; N, 11.71; O, 8.99.

2'-(2-Isobutyl-2*H*-indazol-5-yl)-1',2',5',6',7',7a'-hexahydrospiro[indene-2,3'-pyrrolizine]-1,3-dione (8j)

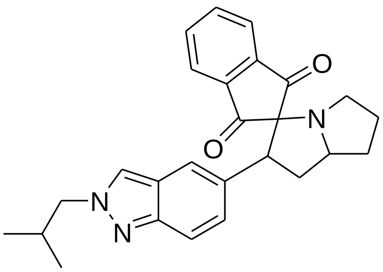

Brown semi solid; yield 0.058g (0.14 mmol, 68%). ^{1}H NMR(400 MHz, MeOD): δ = 7.55-7.76 (5 H, m, 5 CH), 7.39 (1 H, d, ^3J = 0.80 Hz, CH), 7.15 (1 H, d, ^3J = 12.00 Hz, CH), 7.03 (1 H, d, ^3J = 2.00 Hz, CH), 4.16 (1 H, d, ^3J = 28.00 Hz, CH), 3.95 (2 H, t, ^3J = 14.00 Hz, CH_2), 2.86-2.99 (1 H, m, CH), 2.60-2.75 (1 H, m, CH), 2.03-2.10 (3 H, m, 3 CH), 1.55-1.80 (2 H, m, CH_2), 0.75-0.82 (2 H, m, CH_2), 0.61-0.70 (m, 6H). ^{13}C NMR (400 MHz): δ = 21.0, 21.1, 23.7, 25.8, 29.0, 29.8, 32.1, 51.2, 59.4, 61.3, 99.1, 113.5, 120.3, 125.2, 125.8, 129.0, 129.1, 132.5, 133.2, 133.7, 133.9, 137.5, 141.7, 141.9, 198.3, 198.4. LCMS *m/z* (%) = 414.2 [M+1]. Anal. Calcd for $C_{26}H_{27}N_3O_2$ (413.2): C, 75.52; H, 6.58; N, 10.16; O, 7.74. Found: C, 75.66; H, 6.71; N, 10.25; O, 7.91.

2’-(2-Benzyl-2*H*-indazol-5-yl)-1’,2’,5’,6’,7’,7a’-hexahydrospiro[indene-2,3’-pyrrolizine]-1,3-dione (8k)

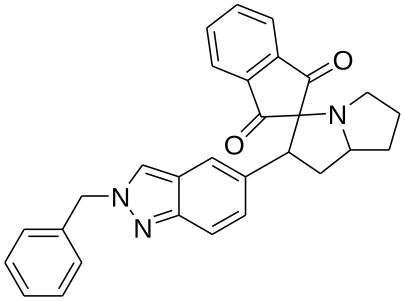

Pale yellow solid; yield 0.047g (0.10 mmol, 68%). ^{1}H NMR (400 MHz, CDCl3) δ = 7.83-7.88 (1 H, m, CH), 7.63-7.74 (4 H, m, 4 CH), 7.40-7.45 (2 H, m, 2 CH), 7.28-7.34 (3 H, m, 3 CH), 7.20-7.24 (2 H, m, 2 CH), 7.02 (1 H, dd, ^{3}J = 2.01, 9.04 Hz, CH), 5.49 (2 H, s, CH_2), 3.85-3.93 (1 H, m, CH), 3.12-3.21 (1 H, m, CH), 2.99 (1 H, ddd, ^{3}J = 8.28, 10.42, 12.42 Hz, CH), 2.77-2.85 (1 H, m, CH), 2.02-2.23 (3 H, m, 3 CH), 1.77-1.91 (3 H, m, 3 CH), 1.57-1.71 (1 H, m, CH). ^{13}C NMR (400 MHz): δ = 23.5, 29.4, 29.6, 32.5, 50.5, 61.8, 62.2, 99.4, 114.9, 120.4, 124.5, 126.4, 126.8, 128.7, 128.8, 128.9, 130.0, 131.7, 133.4, 133.5, 133.8, 136.4, 136.7, 141.8, 141.9, 197.3, 197.5. LCMS *m/z* (%) = 448.2 [M+1]. Anal. Calcd for $C_{29}H_{25}N_3O$ (447.19): C, 77.83; H, 5.63; N, 9.39; O, 7.15. Found: C, 77.83; H, 5.67; N, 9.48; O, 7.23.

References:

(1) Koji, N.; Shinya, T.; Megumi, K.; Satoru, O.; Masafumi, C.; Yuuki, T.; Yosuke, S.; Ryo, O.; Tetsutaro, H. *Tetrahedron,* **2016**, *72*, 734-745.

(2) Boyd, Scott et al. *J. Med. Chem.* **2015**, 58, 3611-3625.

(3) Xiu-Hua Xu.; Guo-Kai liu.; Ayaka, A.; Etsuko, T.; Norio, S. *Org. Lett.* **2011**, 13, 4854-4857.

(4) David, J.S.; Nicholas, F.P.; Wanda, B.; John, W.L.; Paul, S.W. *J. Org. Chem.* 2009, *74,* 6331-6334.

(5) Robert, J.; Phipps.; Neil, P.; Grimster.; Matthew, J. *J. Am. Chem. Soc.* **2008**, 130, 8172-8174.

(6) Crestey, Francois et al. *Synlett*. **2009**, 4, 615-619.

(7) Alan, P.K.; Irina, N.G.; Hongbin, Y.; Pavel, A.P.; Sylvie, Y.B.; Allison, F.; Barbara, C.; Paul, M. *J. Am. Chem. Soc.* **2007**, *129*, 8328-8332.

(8) Starostenko, N. E.; Avdeeva, N. O.; Zeiberlikh, F. N.; Kurkoskaya, L. N.; Suvorov, N. N. *Khimiya Geterotsiklicheskikh Soedinenii*. **1987**, *23*, 325-332.

(9) Gary, A.M.; Adam, R.B. *J. Org. Chem.* **2006**, *71*, 9681-9686.

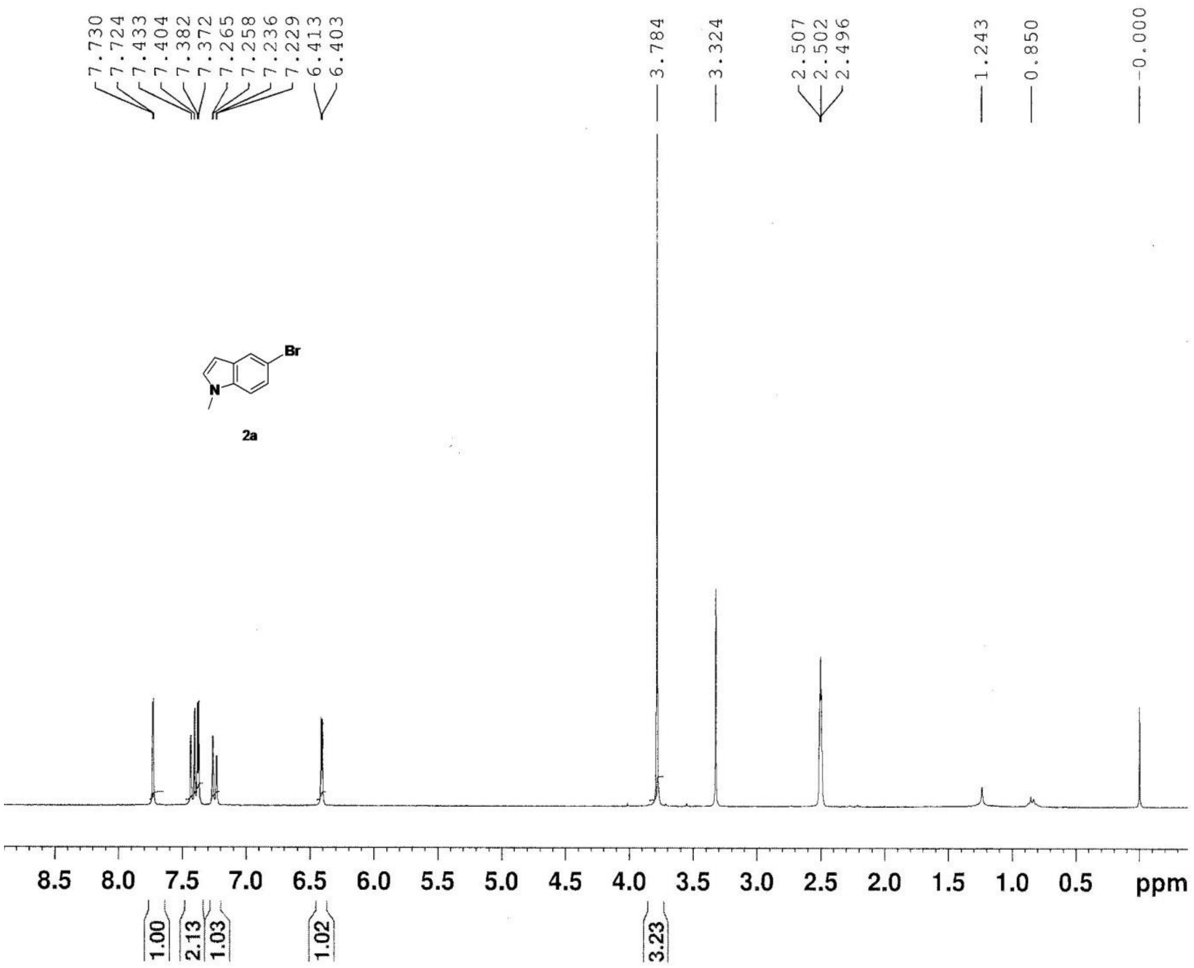
7.730
7.724
7.433
7.404
7.382
7.372
7.265
7.258
7.236
7.229
6.413
6.403
3.784
3.324
2.507
2.502
2.496
1.243
0.850
-0.000
Br
N
2a
8.5 8.0 7.5 7.0 6.5 6.0 5.5 5.0 4.5 4.0 3.5 3.0 2.5 2.0 1.5 1.0 0.5 ppm
1.00
2.13
1.03
1.02
3.23

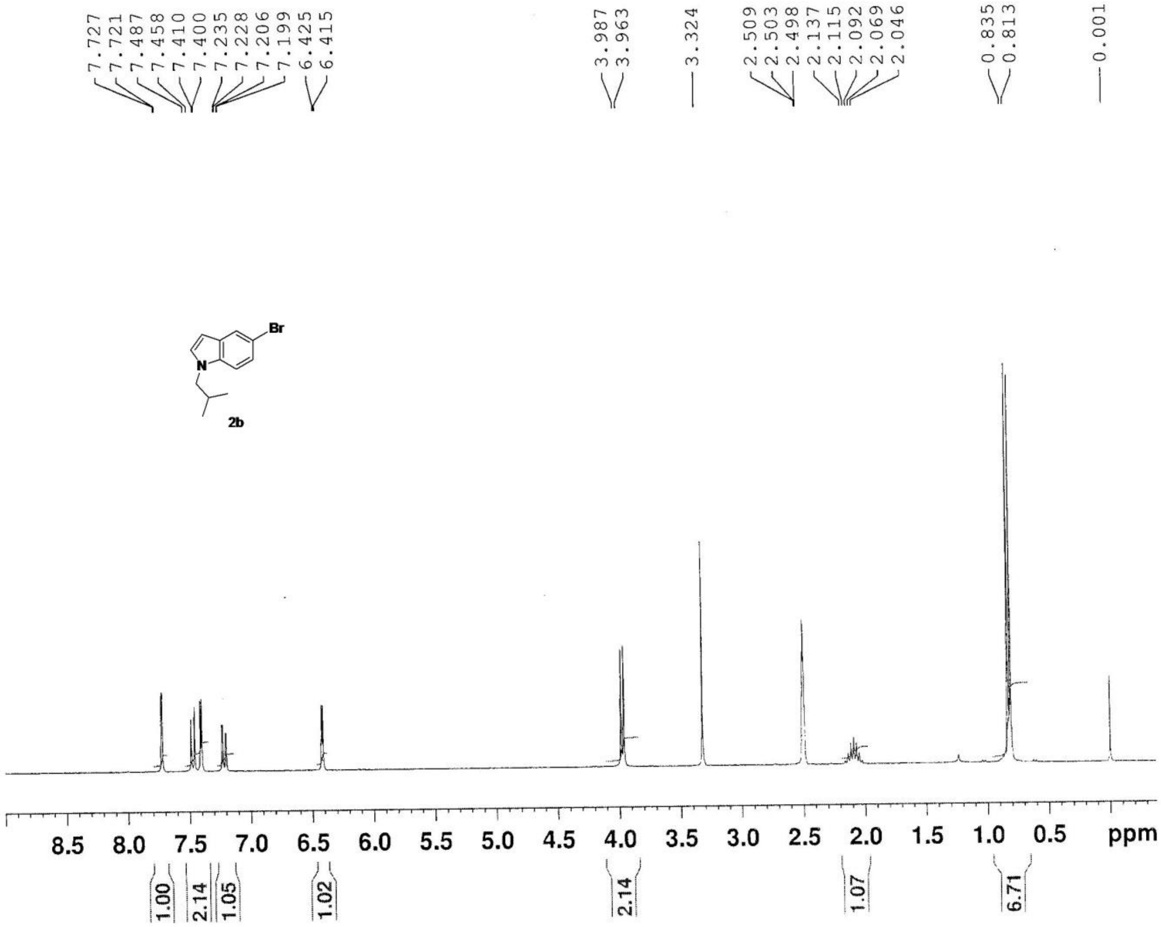

7.727
7.721
7.487
7.458
7.410
7.400
7.235
7.228
7.206
7.199
6.425
6.415
3.987
3.963
3.324
2.509
2.503
2.498
2.137
2.115
2.092
2.069
2.046
0.835
0.813
0.001
Br
N
2b
8.5 8.0 7.5 7.0 6.5 6.0 5.5 5.0 4.5 4.0 3.5 3.0 2.5 2.0 1.5 1.0 0.5 ppm
1.00
2.14
1.05
1.02
2.14
1.07
6.71

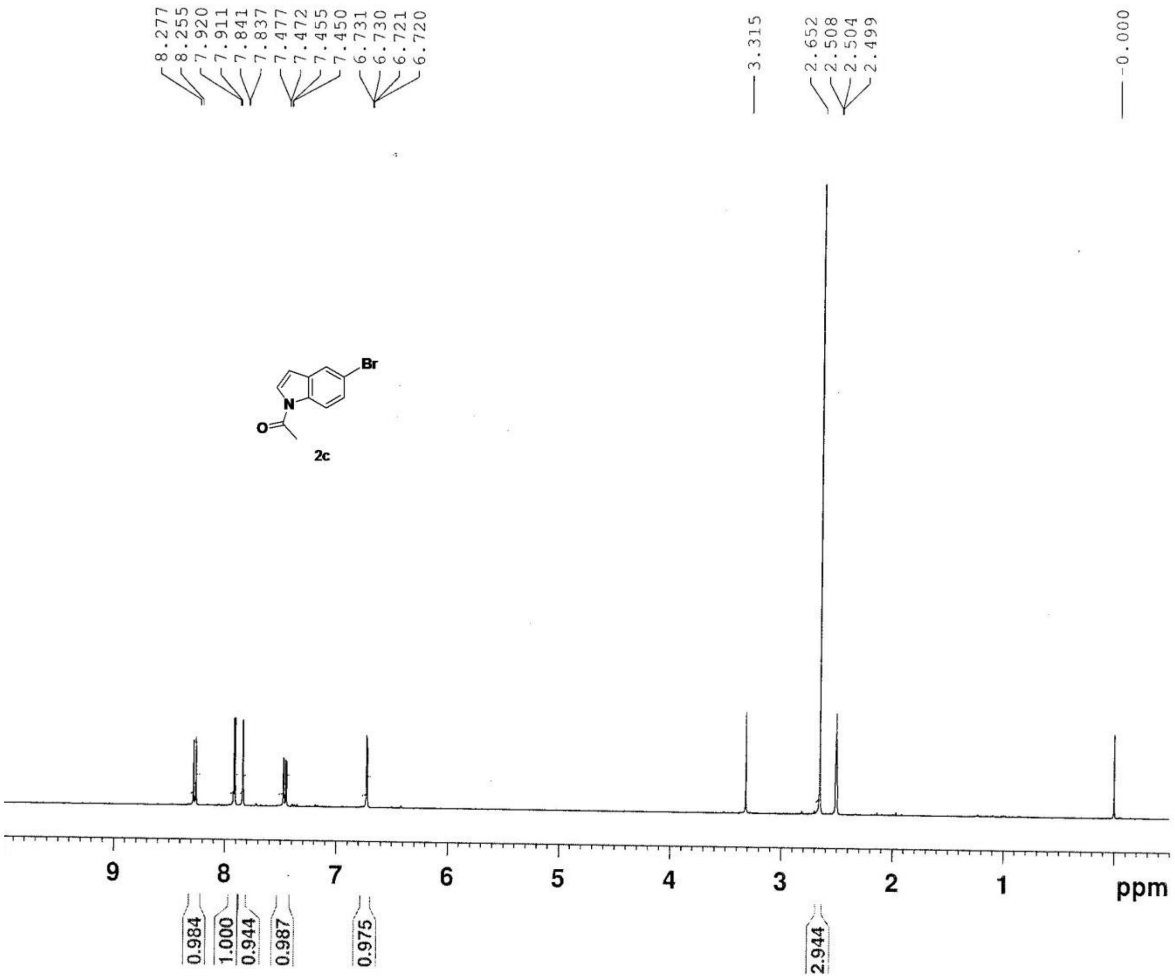
8.277
8.255
7.920
7.911
7.841
7.837
7.477
7.472
7.455
7.450
6.731
6.730
6.721
6.720
3.315
2.652
2.508
2.504
2.499
-0.000
Br
N
O
2c
9
8
7
6
5
4
3
2
1
ppm
0.984
1.000
0.944
0.987
0.975
2.944

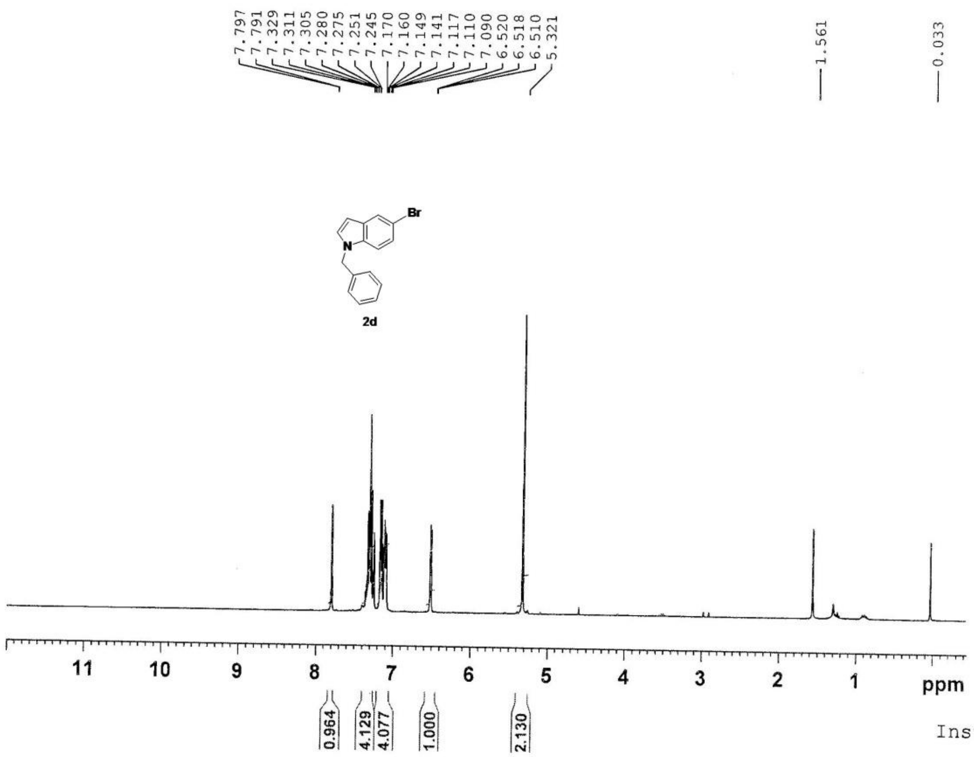

Instrument ID: AV-300

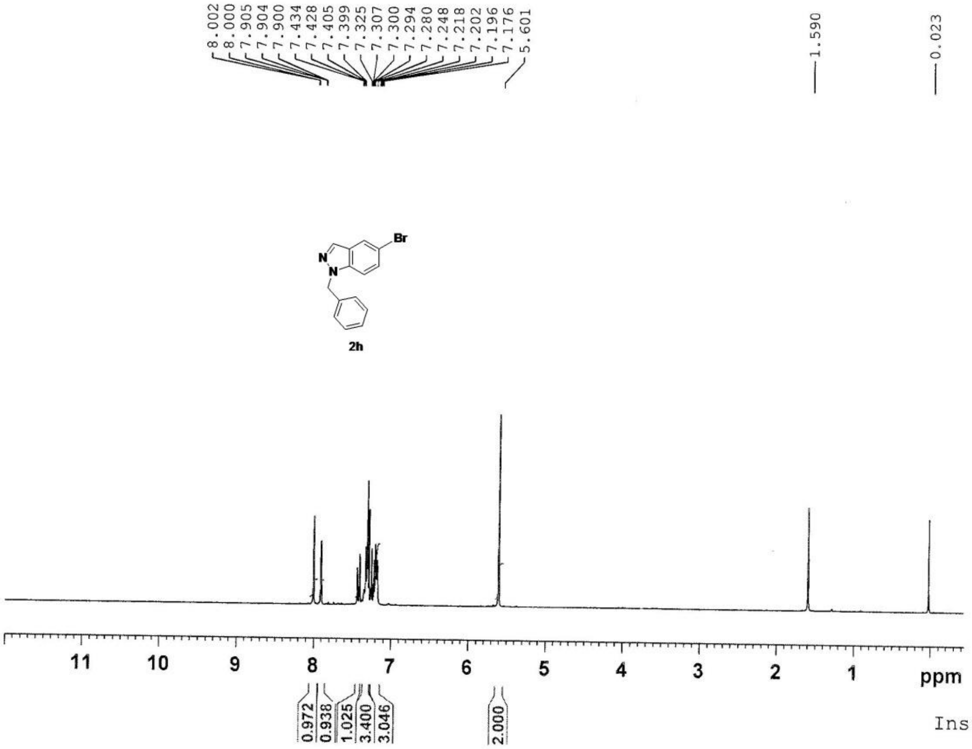

Instrument ID: AV-300

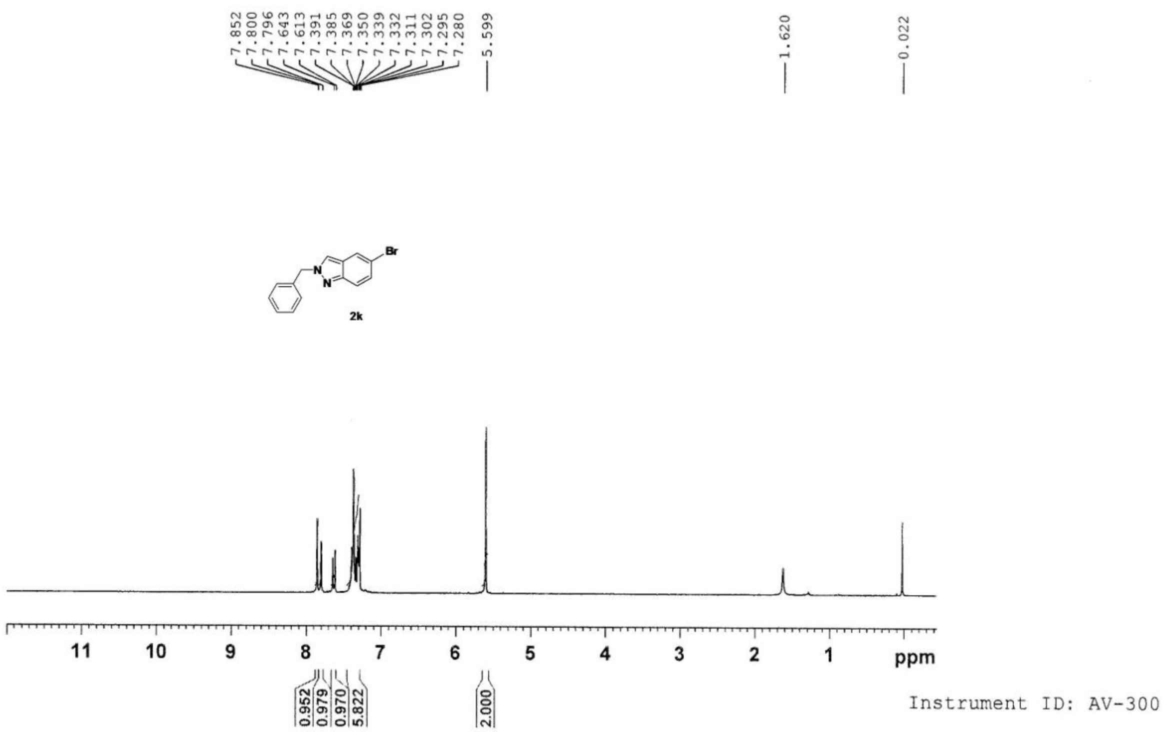
7.852
7.800
7.796
7.643
7.613
7.391
7.385
7.369
7.350
7.339
7.332
7.311
7.302
7.295
7.280
5.599
1.620
0.022
Br
N
N
2k
11
10
9
8
7
6
5
4
3
2
1
ppm
0.952
0.979
0.970
5.822
2.000
Instrument ID: AV-300

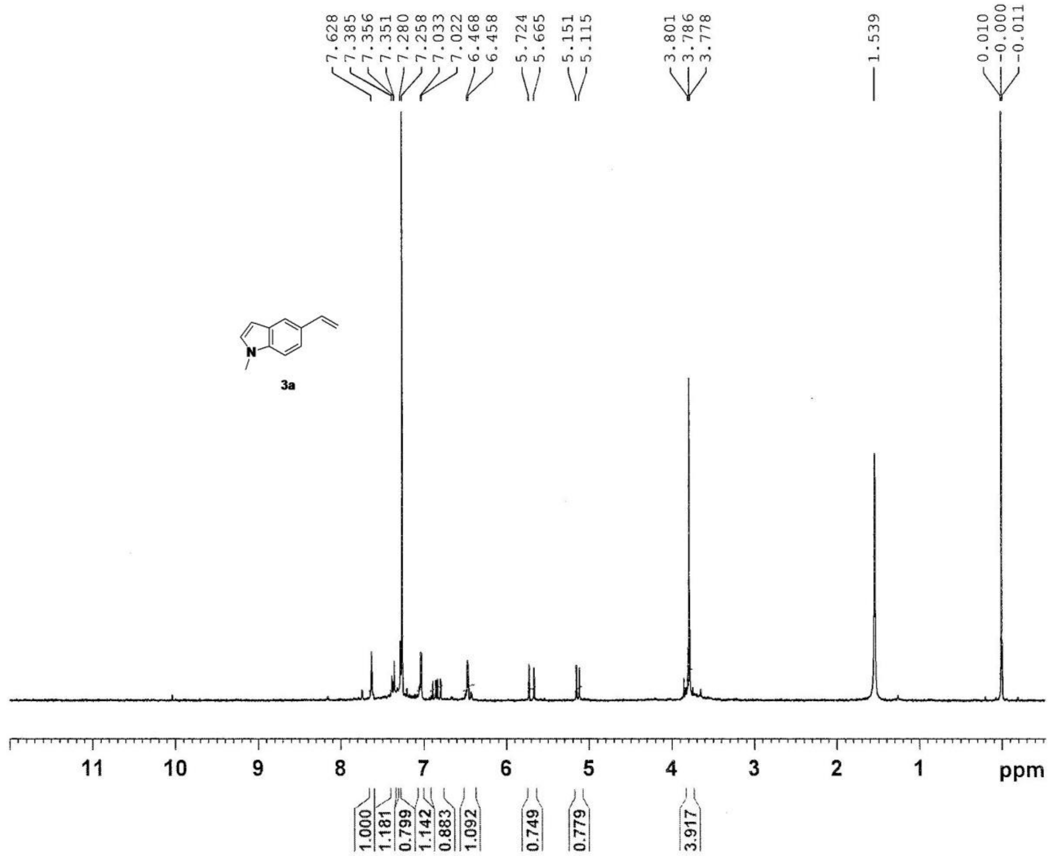

Instrument ID: AV-300

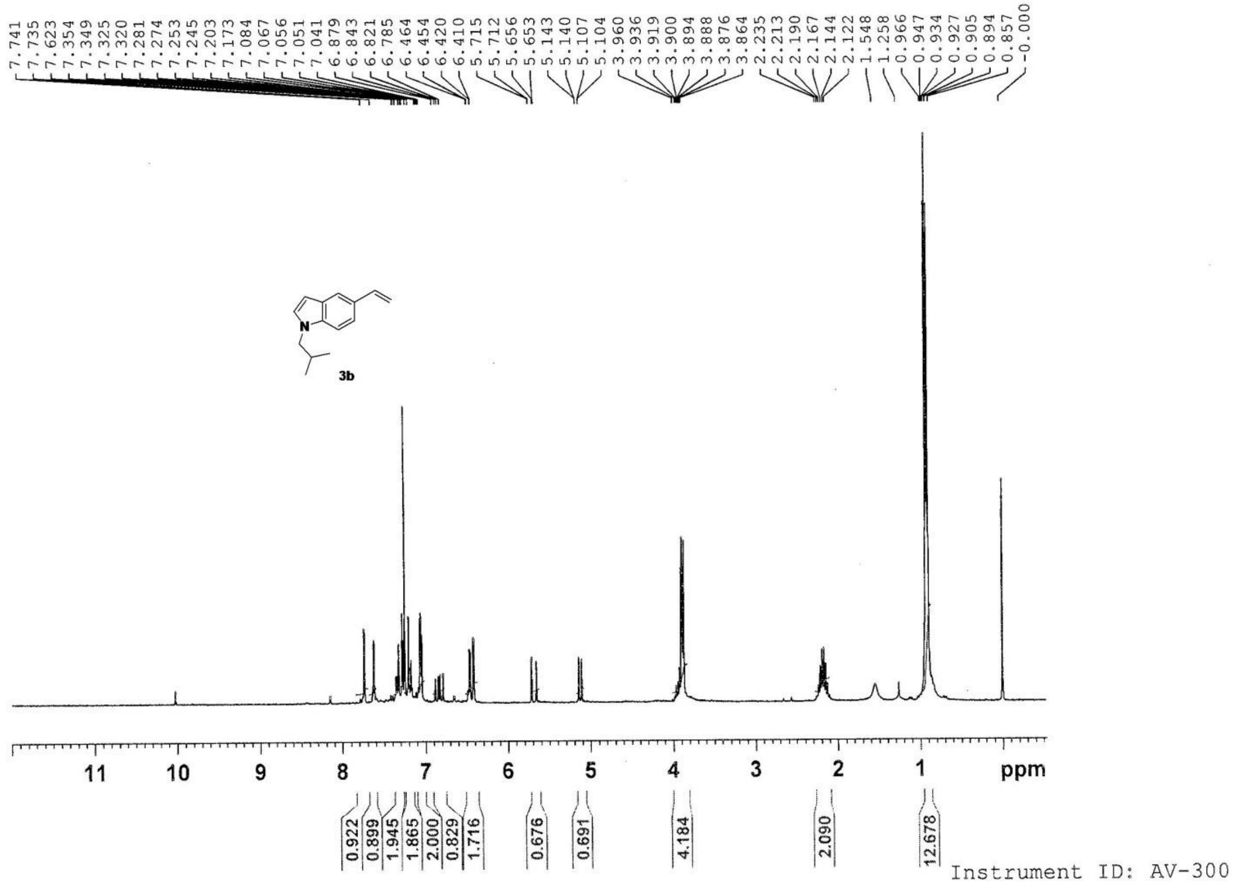
7.741
7.735
7.623
7.354
7.349
7.325
7.320
7.281
7.274
7.253
7.245
7.203
7.173
7.084
7.067
7.056
7.051
7.041
6.879
6.843
6.821
6.785
6.464
6.454
6.420
6.410
5.715
5.712
5.656
5.653
5.143
5.140
5.107
5.104
3.960
3.936
3.919
3.900
3.894
3.888
3.876
3.864
2.235
2.213
2.190
2.167
2.144
2.122
1.548
1.258
0.966
0.947
0.934
0.927
0.905
0.894
0.857
-0.000
N
3b
11
10
9
8
7
6
5
4
3
2
1
ppm
0.922
0.899
1.945
1.865
2.000
0.829
1.716
0.676
0.691
4.184
2.090
12.678
Instrument ID: AV-300

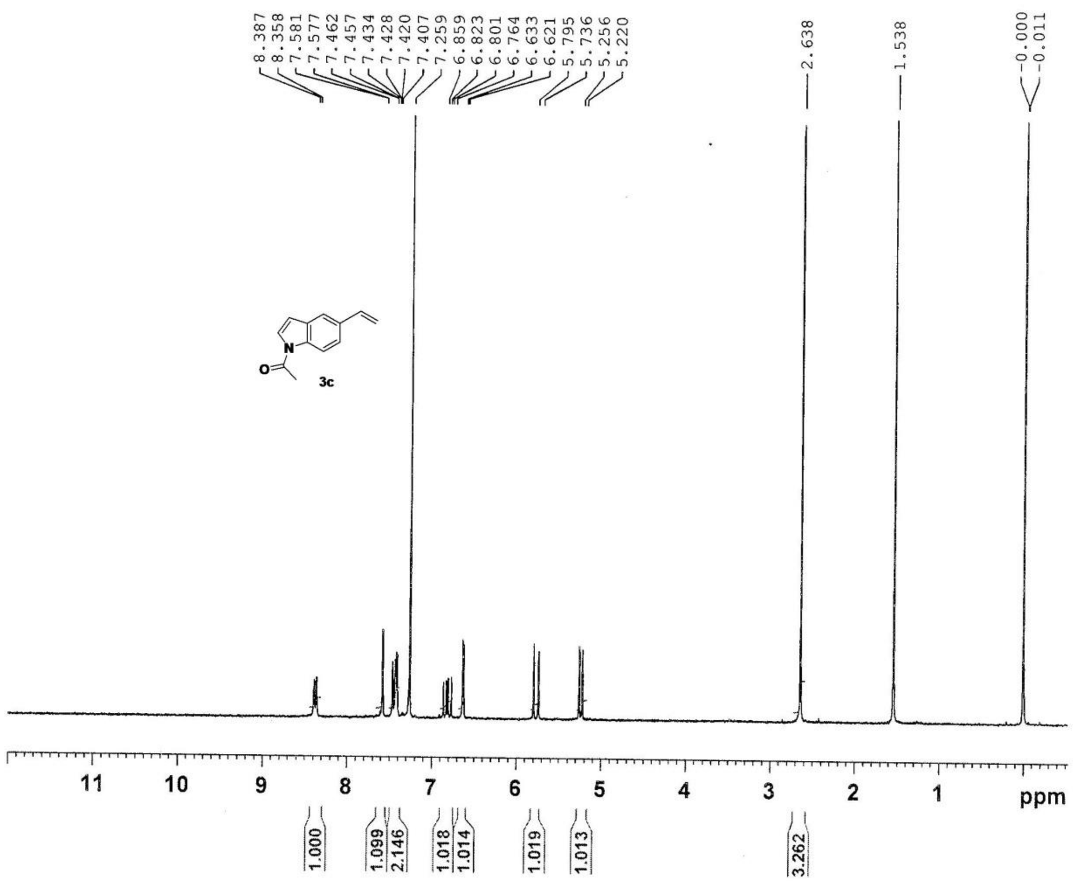

Instrument ID: AV-300

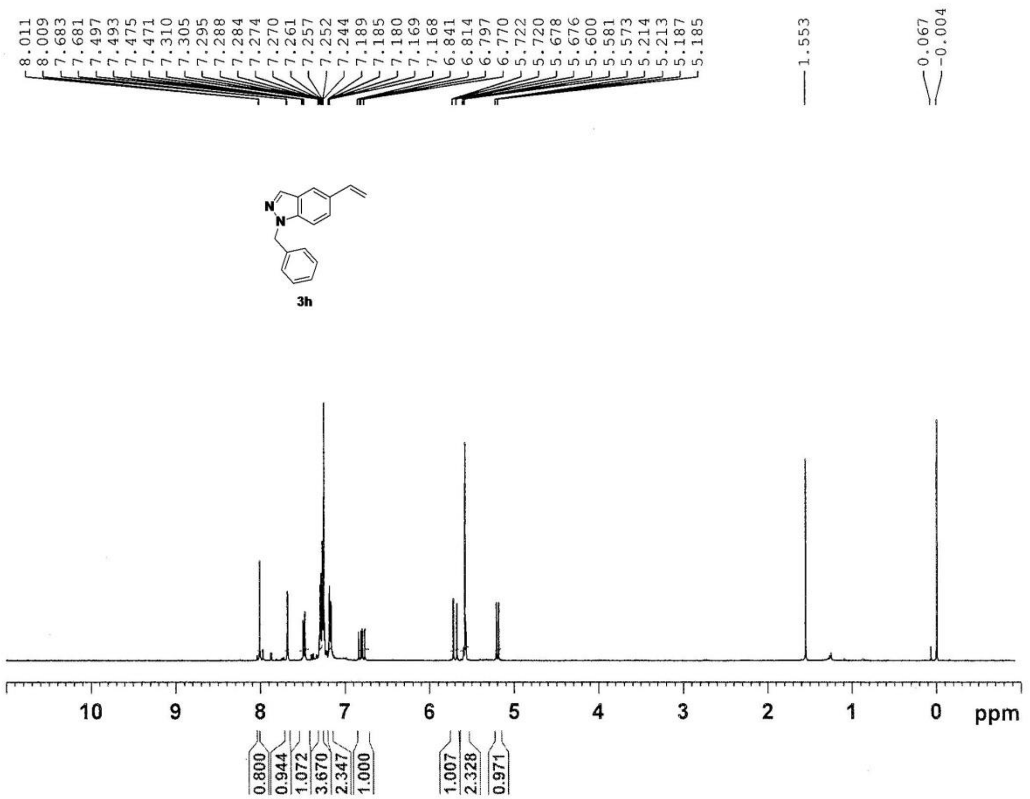

3h

Instrument ID: AV-400-11

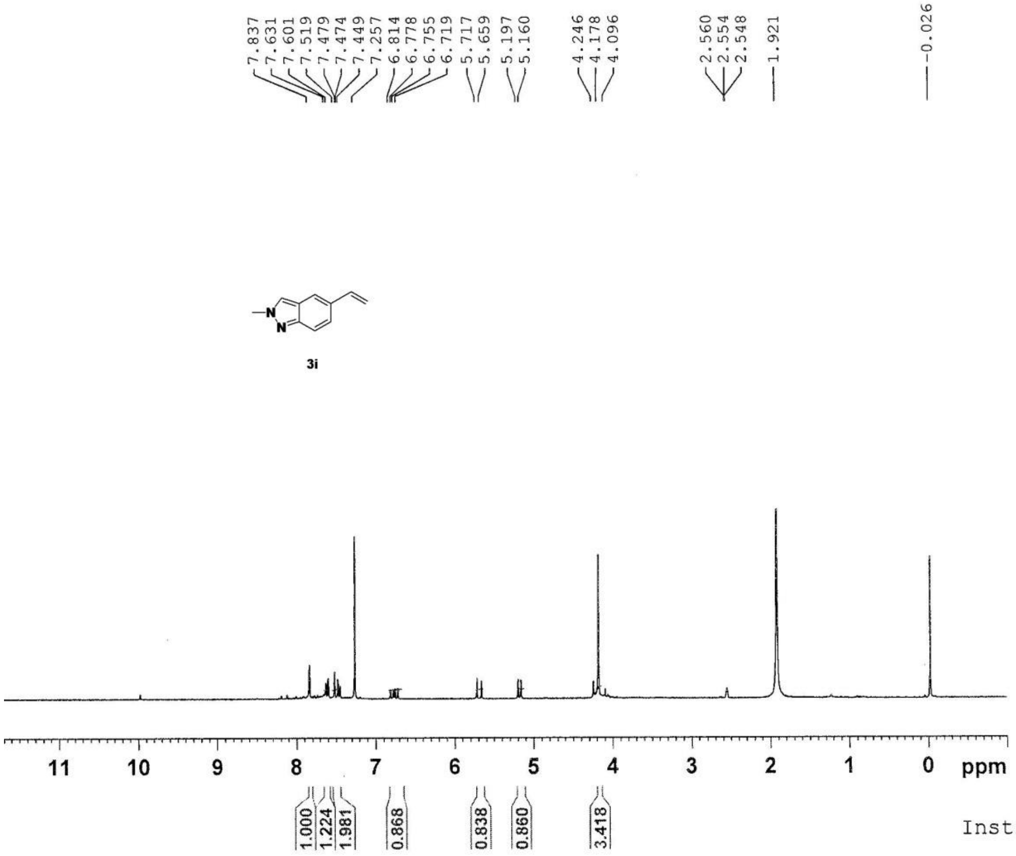

Instrument ID: AV-300

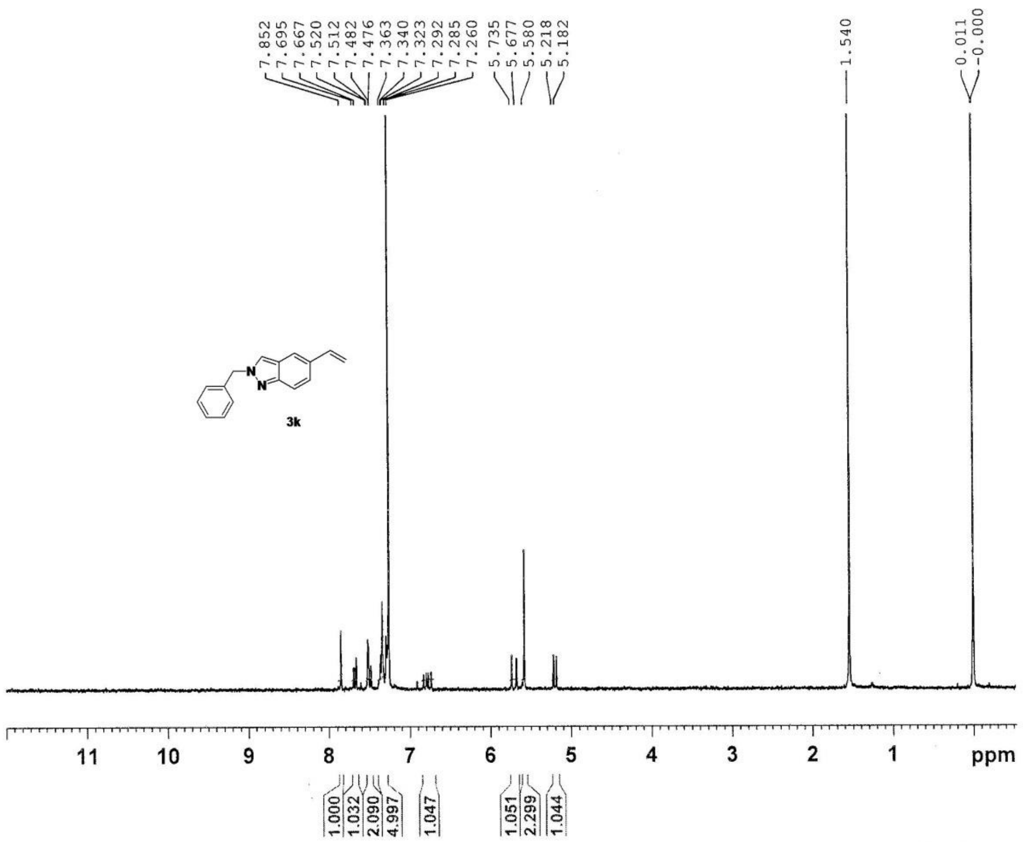

Instrument ID: AV-300

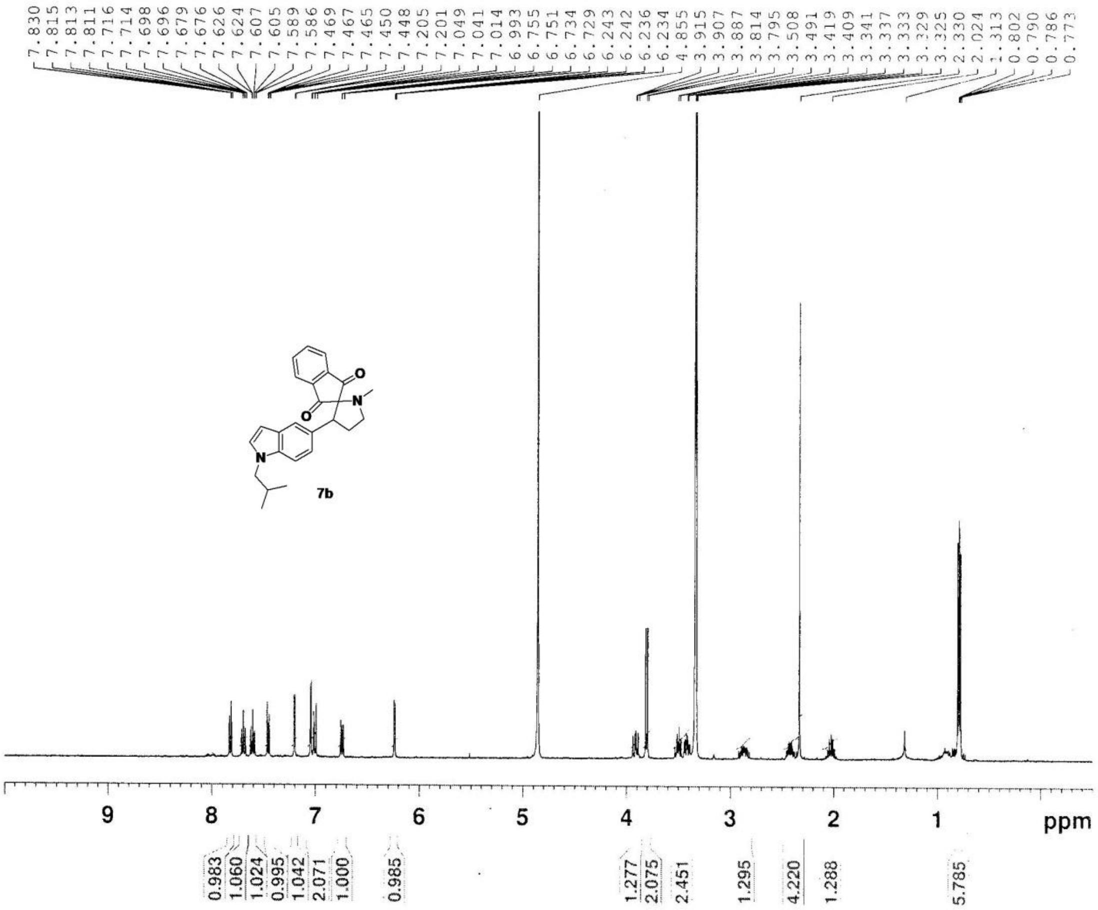
7.830
7.815
7.813
7.811
7.716
7.714
7.698
7.696
7.679
7.676
7.626
7.624
7.607
7.605
7.589
7.586
7.469
7.467
7.465
7.450
7.448
7.205
7.201
7.049
7.041
7.014
6.993
6.755
6.751
6.734
6.729
6.243
6.242
6.236
6.234
4.855
3.915
3.907
3.887
3.814
3.795
3.508
3.491
3.419
3.409
3.341
3.337
3.333
3.329
3.325
2.330
2.024
1.313
0.802
0.790
0.786
0.773
O
O
N
N
7b
9
8
7
6
5
4
3
2
1
ppm
0.983
1.060
1.024
0.995
1.042
2.071
1.000
0.985
1.277
2.075
2.451
1.295
4.220
1.288
5.785

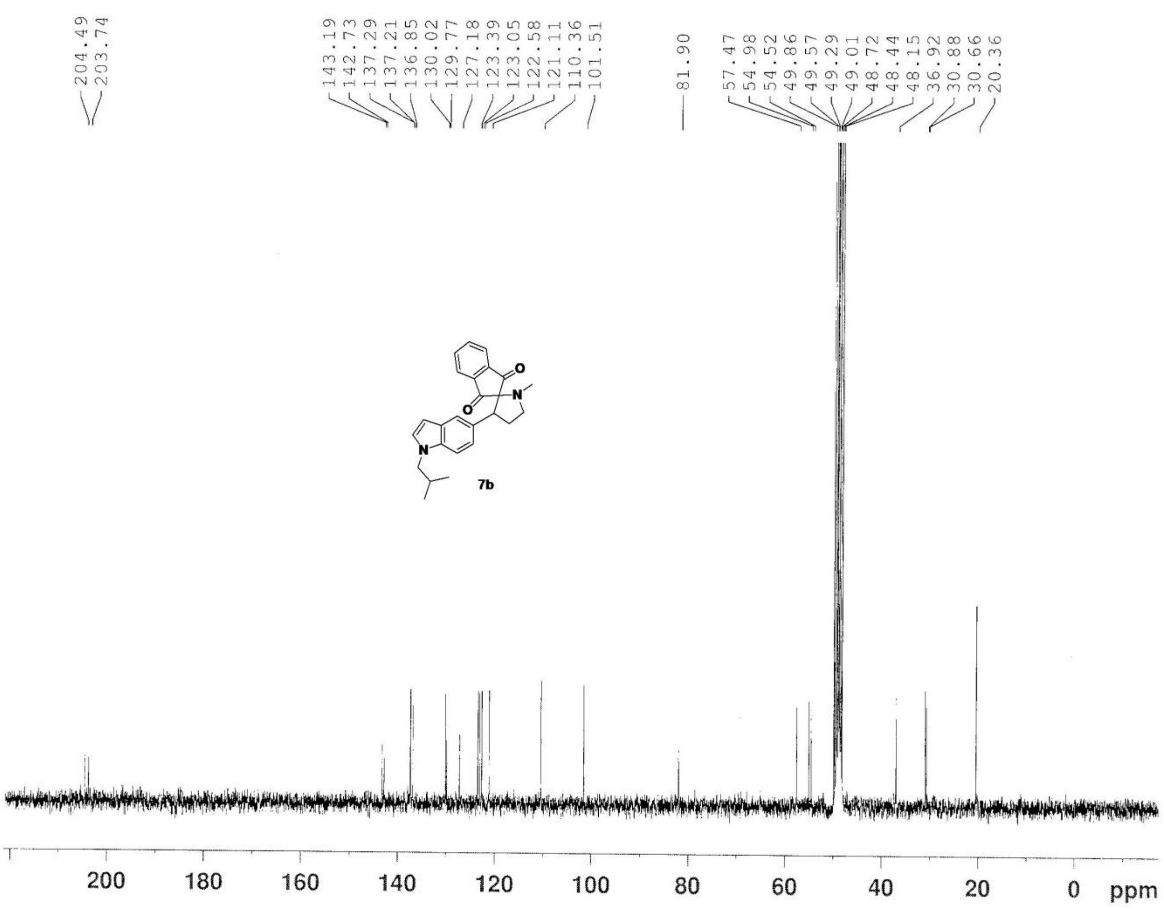

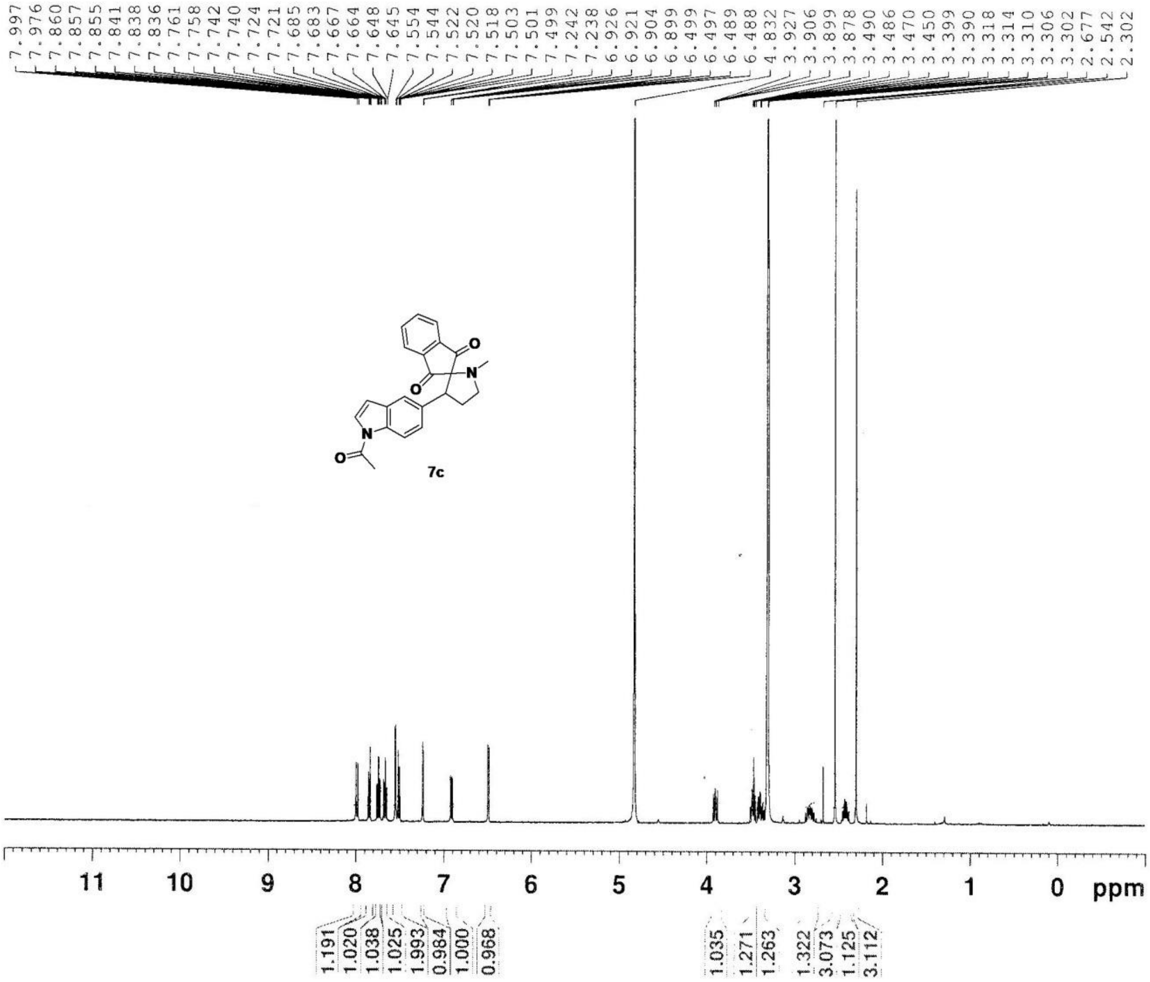

7.997
7.976
7.860
7.857
7.855
7.841
7.838
7.836
7.761
7.758
7.742
7.740
7.724
7.721
7.685
7.683
7.667
7.664
7.648
7.645
7.554
7.544
7.522
7.520
7.518
7.503
7.501
7.499
7.242
7.238
6.926
6.921
6.904
6.899
6.499
6.497
6.489
6.488
4.832
3.927
3.906
3.899
3.878
3.490
3.486
3.470
3.450
3.399
3.390
3.318
3.314
3.310
3.306
3.302
2.677
2.542
2.302
O
N
O
N
O
7c
11
10
9
8
7
6
5
4
3
2
1
0
ppm
1.191
1.020
1.038
1.025
1.993
0.984
1.000
0.968
1.035
1.271
1.263
1.322
3.073
1.125
3.112

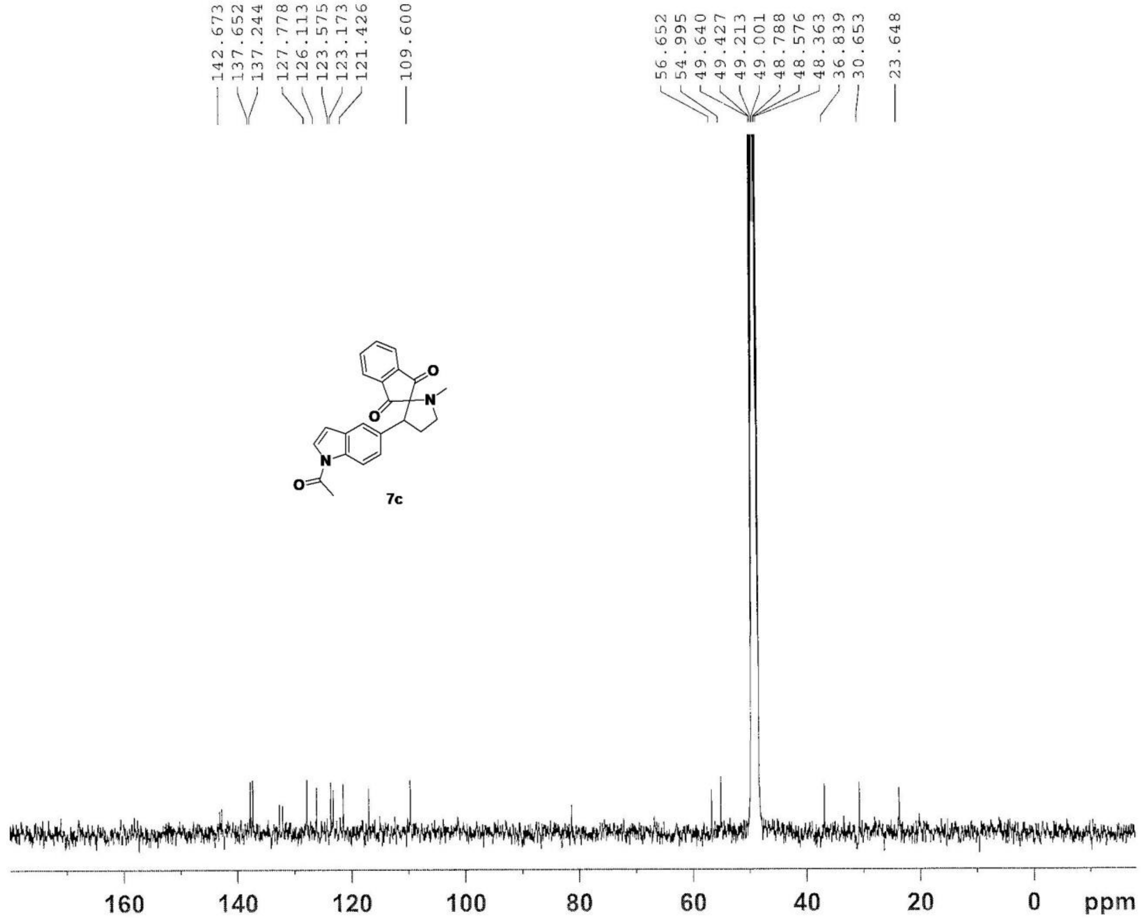
142.673
137.652
137.244
127.778
126.113
123.575
123.173
121.426
109.600
56.652
54.995
49.640
49.427
49.213
49.001
48.788
48.576
48.363
36.839
30.653
23.648
7c
160
140
120
100
80
60
40
20
0
ppm

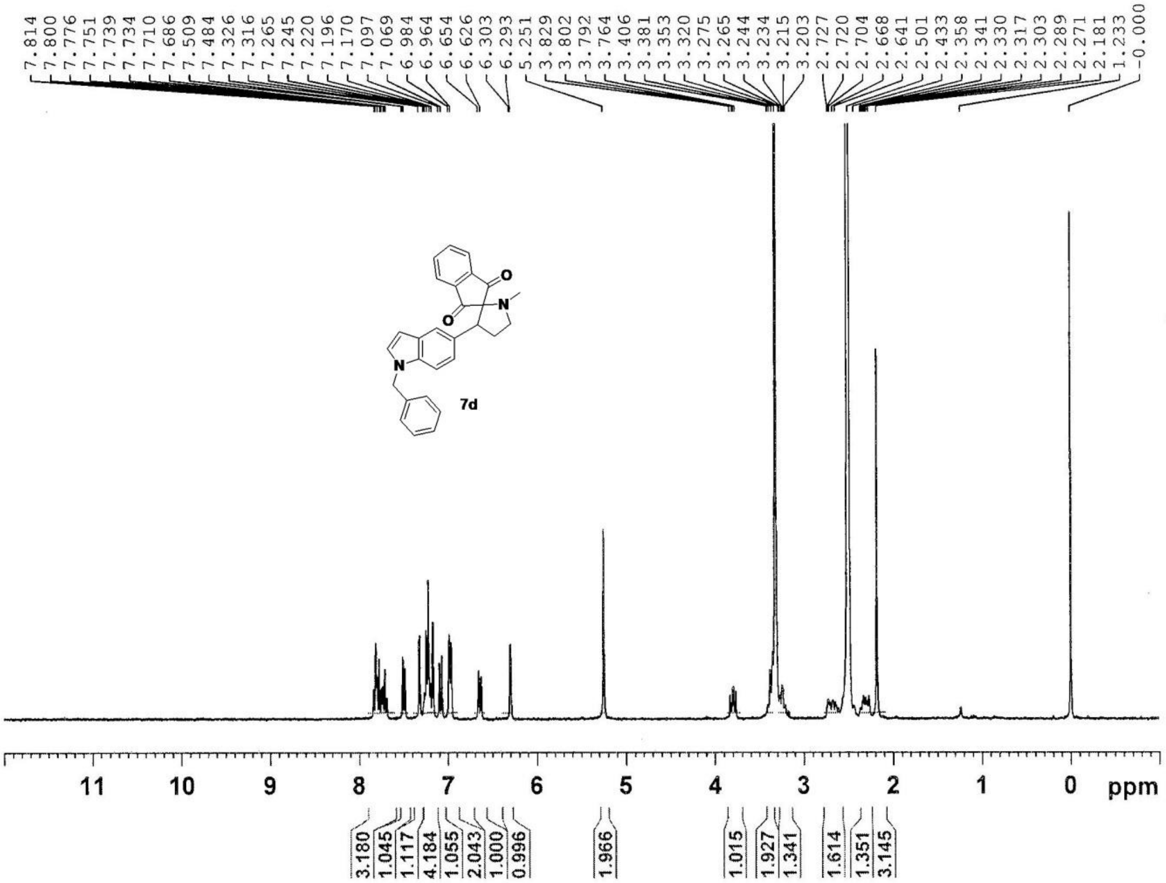
7.814 7.800 7.776 7.751 7.739 7.734 7.710 7.686 7.509 7.484 7.326 7.316 7.265 7.245 7.220 7.196 7.170 7.097 7.069 6.984 6.964 6.654 6.626 6.303 6.293 5.251 3.829 3.802 3.792 3.764 3.406 3.381 3.353 3.320 3.275 3.265 3.244 3.234 3.215 3.203 2.727 2.720 2.704 2.668 2.641 2.501 2.433 2.358 2.341 2.330 2.317 2.303 2.289 2.271 2.181 1.233 -0.000
O
O
N
N
7d
11 10 9 8 7 6 5 4 3 2 1 0 ppm
3.180 1.045 1.117 4.184 1.055 2.043 1.000 0.996 1.966 1.015 1.927 1.341 1.614 1.351 3.145

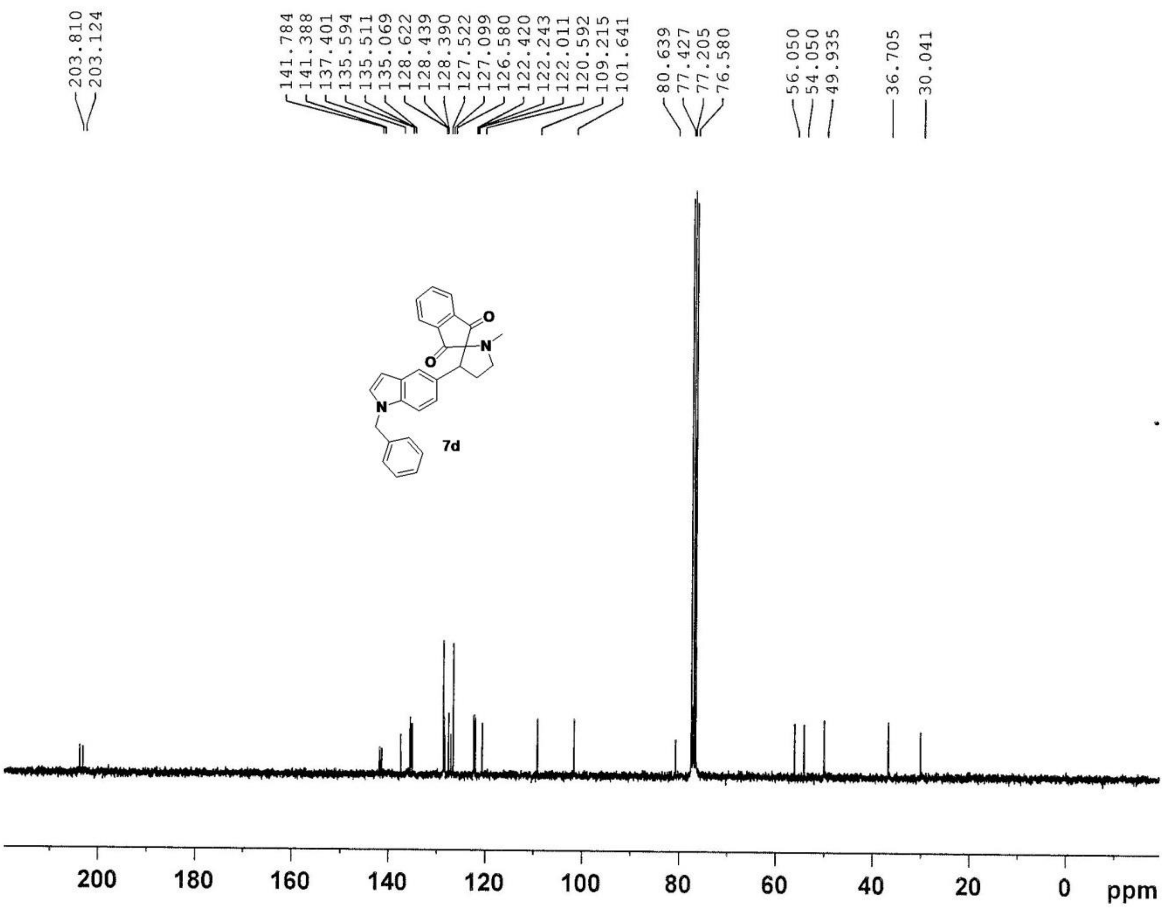
203.810
203.124
141.784
141.388
137.401
135.594
135.511
135.069
128.622
128.439
128.390
127.522
127.099
126.580
122.420
122.243
122.011
120.592
109.215
101.641
80.639
77.427
77.205
76.580
56.050
54.050
49.935
36.705
30.041
7d
200
180
160
140
120
100
80
60
40
20
0
ppm

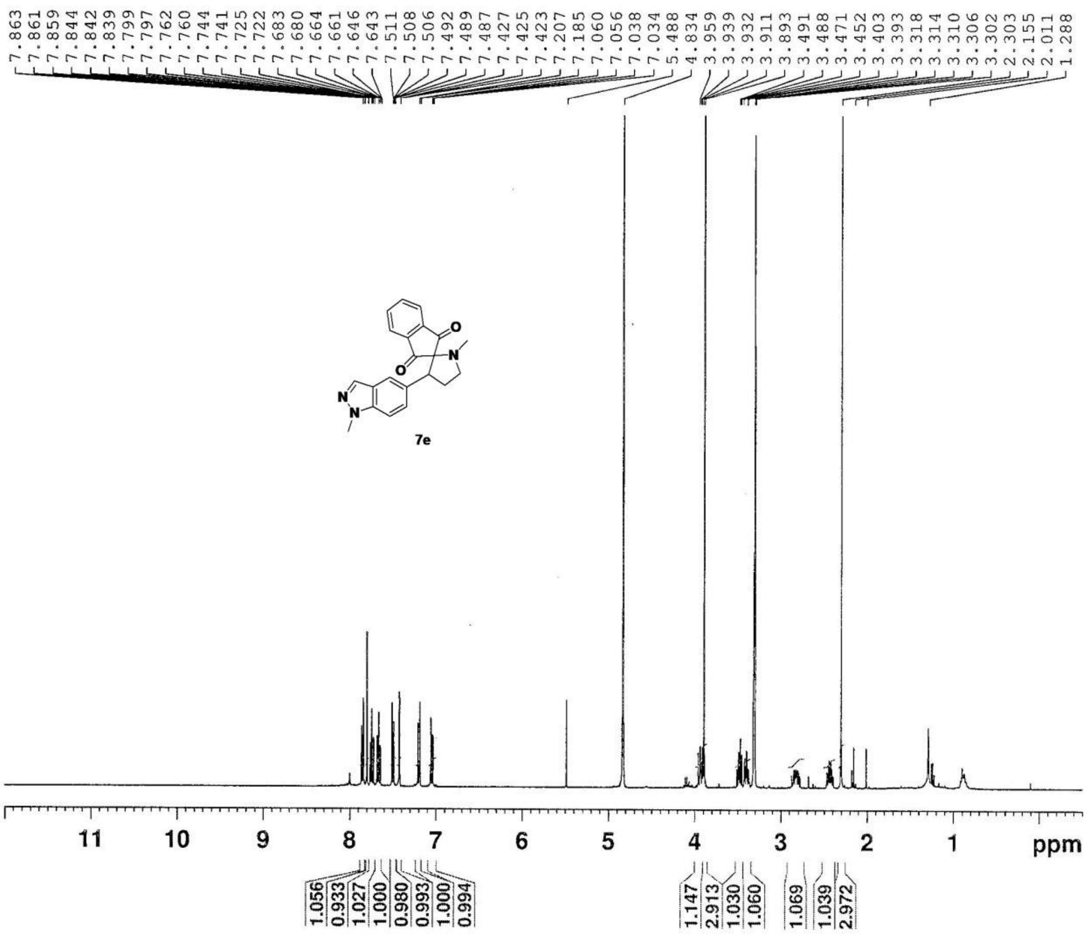
7.863
7.861
7.859
7.844
7.842
7.839
7.799
7.797
7.762
7.760
7.744
7.741
7.725
7.722
7.683
7.680
7.664
7.661
7.646
7.643
7.511
7.508
7.506
7.492
7.489
7.487
7.427
7.425
7.423
7.207
7.185
7.060
7.056
7.038
7.034
5.488
4.834
3.959
3.939
3.932
3.911
3.893
3.491
3.488
3.471
3.452
3.403
3.393
3.318
3.314
3.310
3.306
3.302
2.303
2.155
2.011
1.288
O
O
N
N
N
7e
11
10
9
8
7
6
5
4
3
2
1
ppm
1.056
0.933
1.027
1.000
0.980
0.993
1.000
0.994
1.147
2.913
1.030
1.060
1.069
1.039
2.972

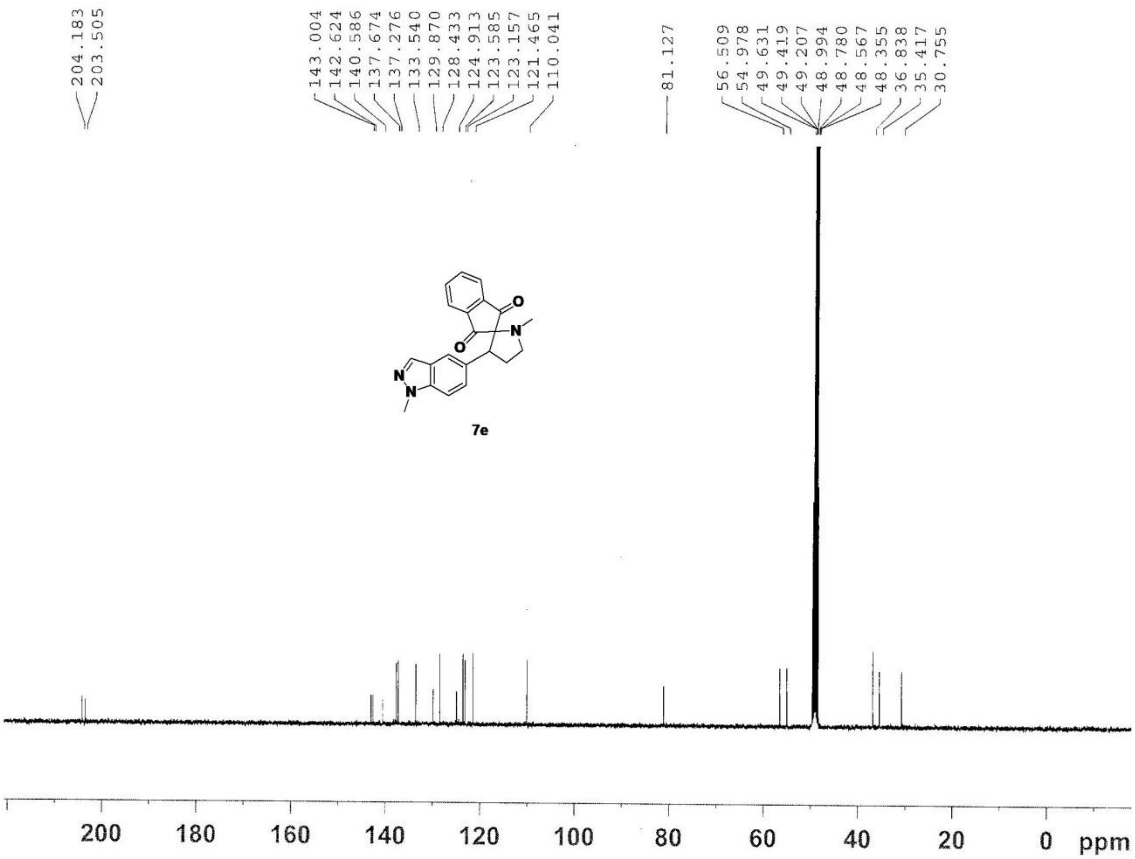
204.183
203.505
143.004
142.624
140.586
137.674
137.276
133.540
129.870
128.433
124.913
123.585
123.157
121.465
110.041
81.127
56.509
54.978
49.631
49.419
49.207
48.994
48.780
48.567
48.355
36.838
35.417
30.755
7e
200 180 160 140 120 100 80 60 40 20 0 ppm

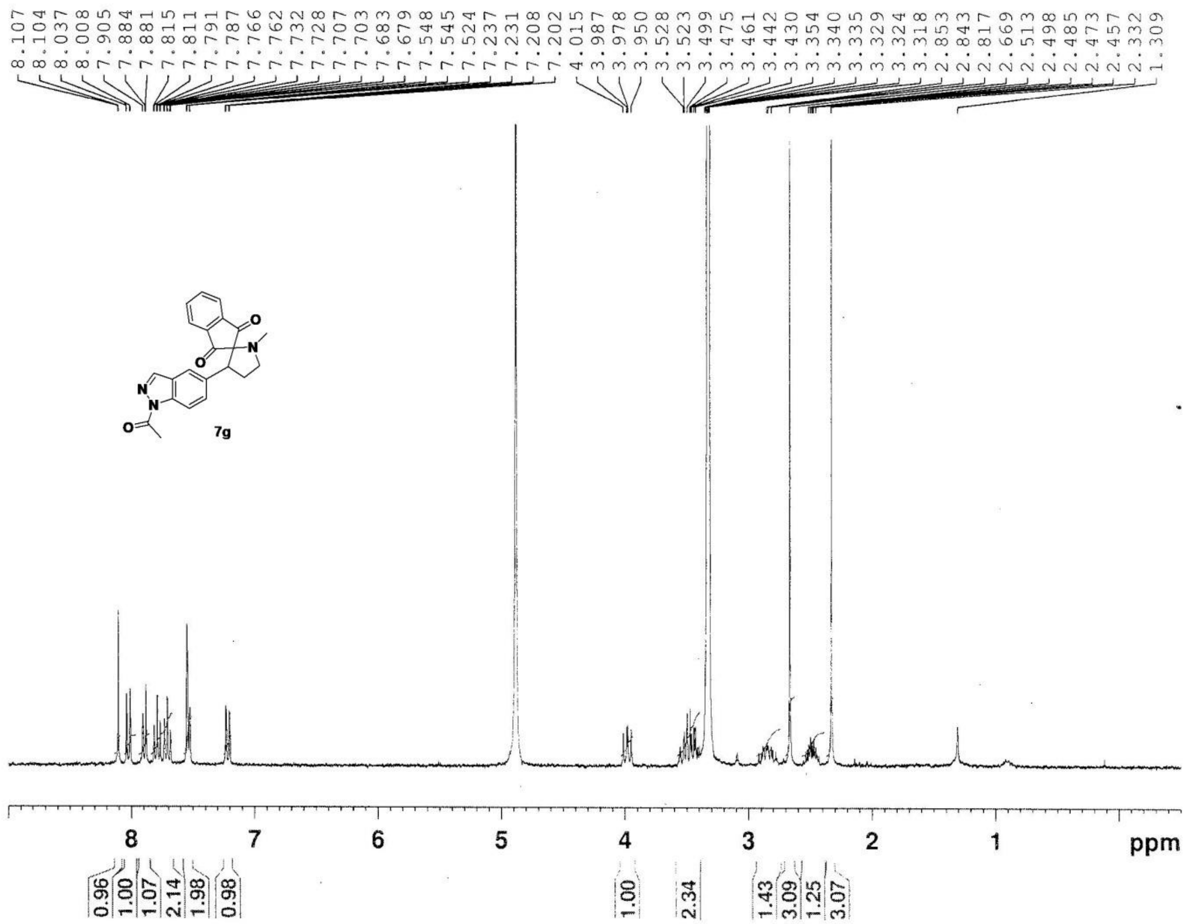
8.107
8.104
8.037
8.008
7.905
7.884
7.881
7.815
7.811
7.791
7.787
7.766
7.762
7.732
7.728
7.707
7.703
7.683
7.679
7.548
7.545
7.524
7.237
7.231
7.208
7.202
4.015
3.987
3.978
3.950
3.528
3.523
3.499
3.475
3.461
3.442
3.430
3.354
3.340
3.335
3.329
3.324
3.318
2.853
2.843
2.817
2.669
2.513
2.498
2.485
2.473
2.457
2.332
1.309
7g
ppm
0.96
1.00
1.07
2.14
1.98
0.98
1.00
2.34
1.43
3.09
1.25
3.07

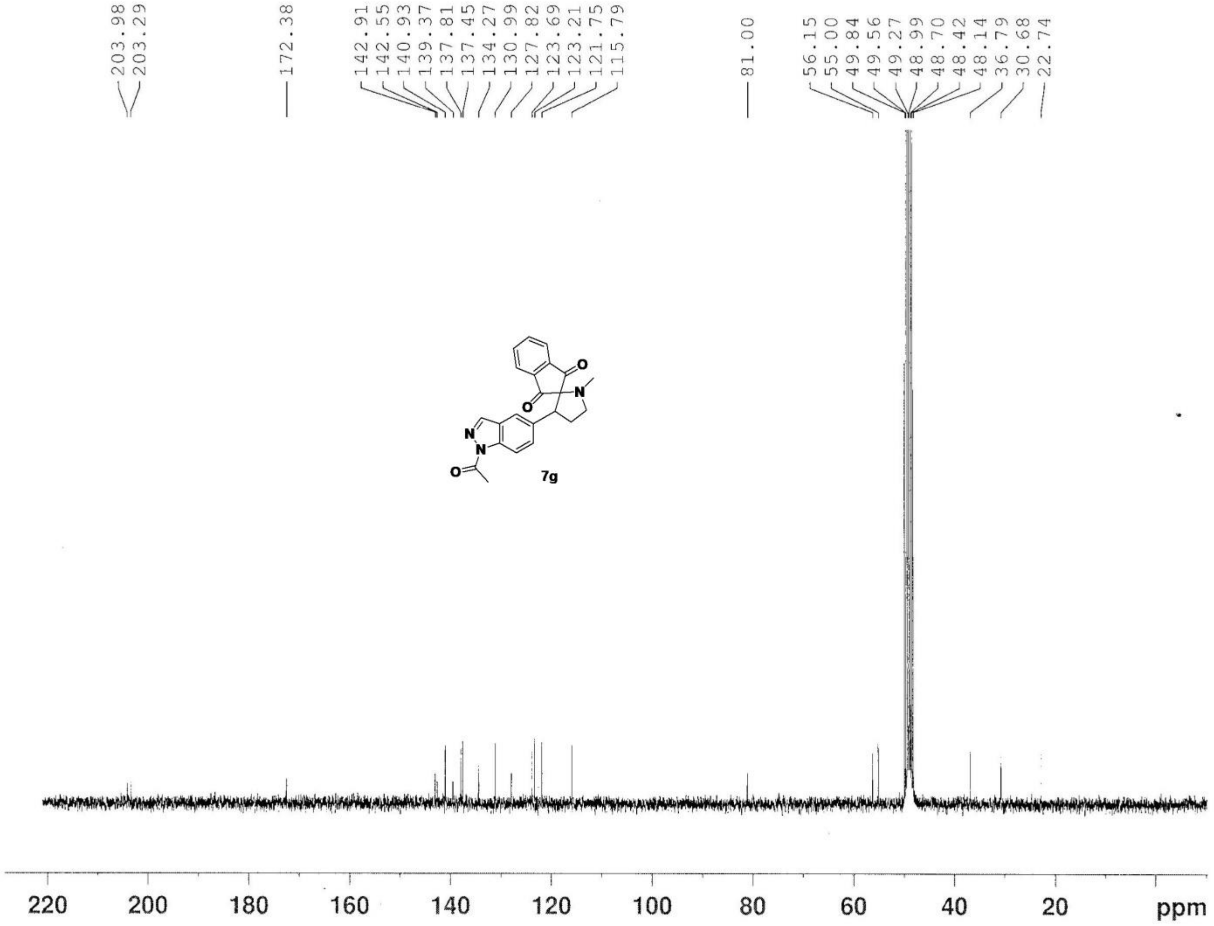
203.98
203.29
172.38
142.91
142.55
140.93
139.37
137.81
137.45
134.27
130.99
127.82
123.69
123.21
121.75
115.79
81.00
56.15
55.00
49.84
49.56
49.27
48.99
48.70
48.42
48.14
36.79
30.68
22.74
O
N
O
N
N
O
7g
220
200
180
160
140
120
100
80
60
40
20
ppm

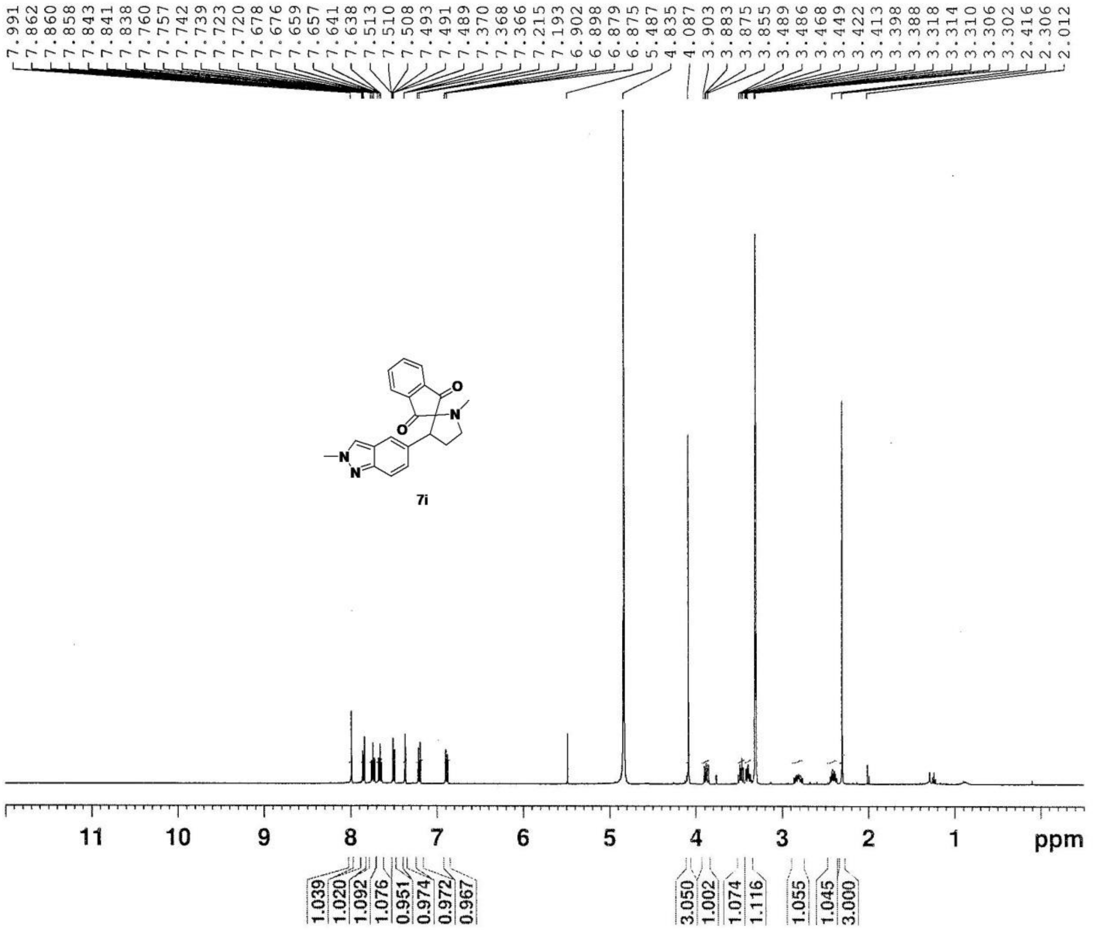
7.991 7.862 7.860 7.858 7.843 7.841 7.838 7.760 7.757 7.742 7.739 7.723 7.720 7.678 7.676 7.659 7.657 7.641 7.638 7.513 7.510 7.508 7.493 7.491 7.489 7.370 7.368 7.366 7.215 7.193 6.902 6.898 6.879 6.875 5.487 4.835 4.087 3.903 3.883 3.875 3.855 3.489 3.486 3.468 3.449 3.422 3.413 3.398 3.388 3.318 3.314 3.310 3.306 3.302 2.416 2.306 2.012
7i
11 10 9 8 7 6 5 4 3 2 1 ppm
1.039 1.020 1.092 1.076 0.951 0.974 0.972 0.967
3.050 1.002 1.074 1.116 1.055 1.045 3.000

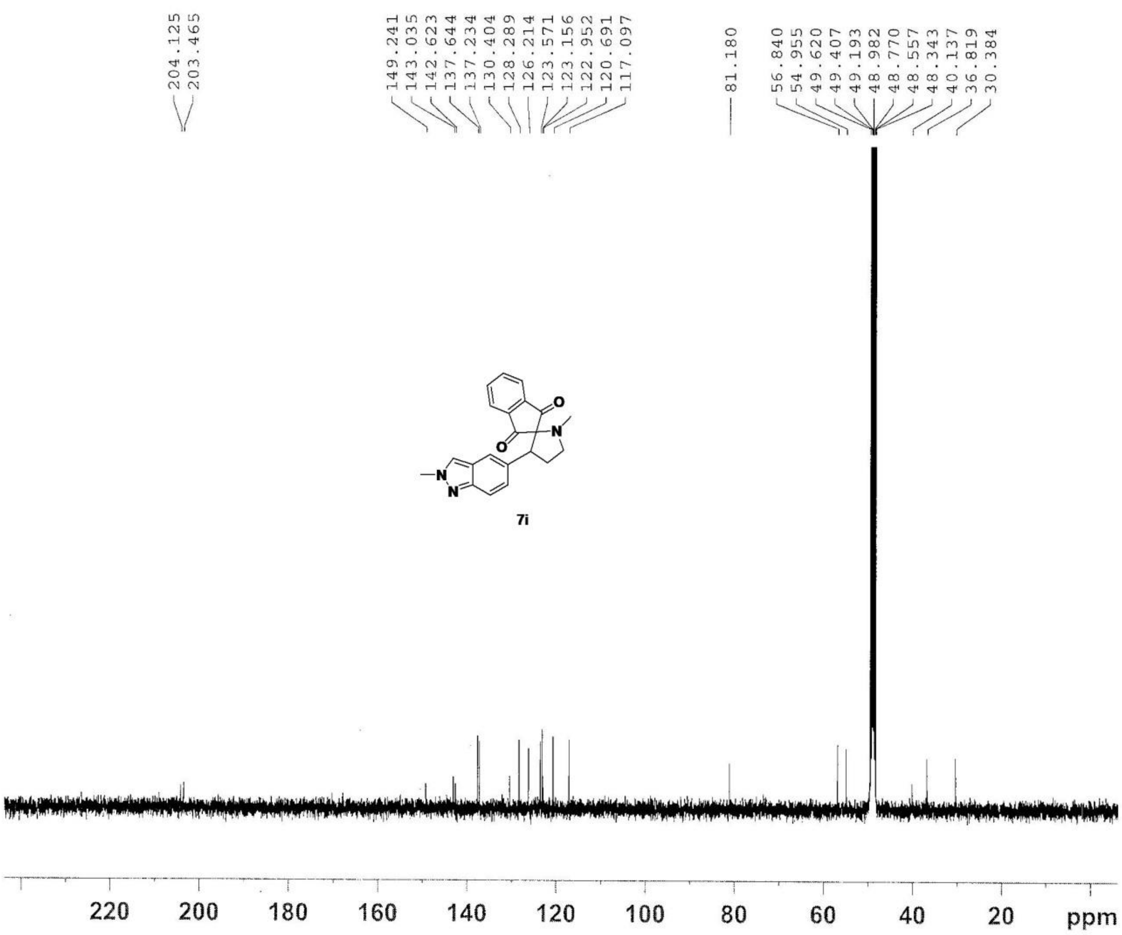
204.125
203.465
149.241
143.035
142.623
137.644
137.234
130.404
128.289
126.214
123.571
123.156
122.952
120.691
117.097
81.180
56.840
54.955
49.620
49.407
49.193
48.982
48.770
48.557
48.343
40.137
36.819
30.384
O
N
O
N
N
7i
220
200
180
160
140
120
100
80
60
40
20
ppm

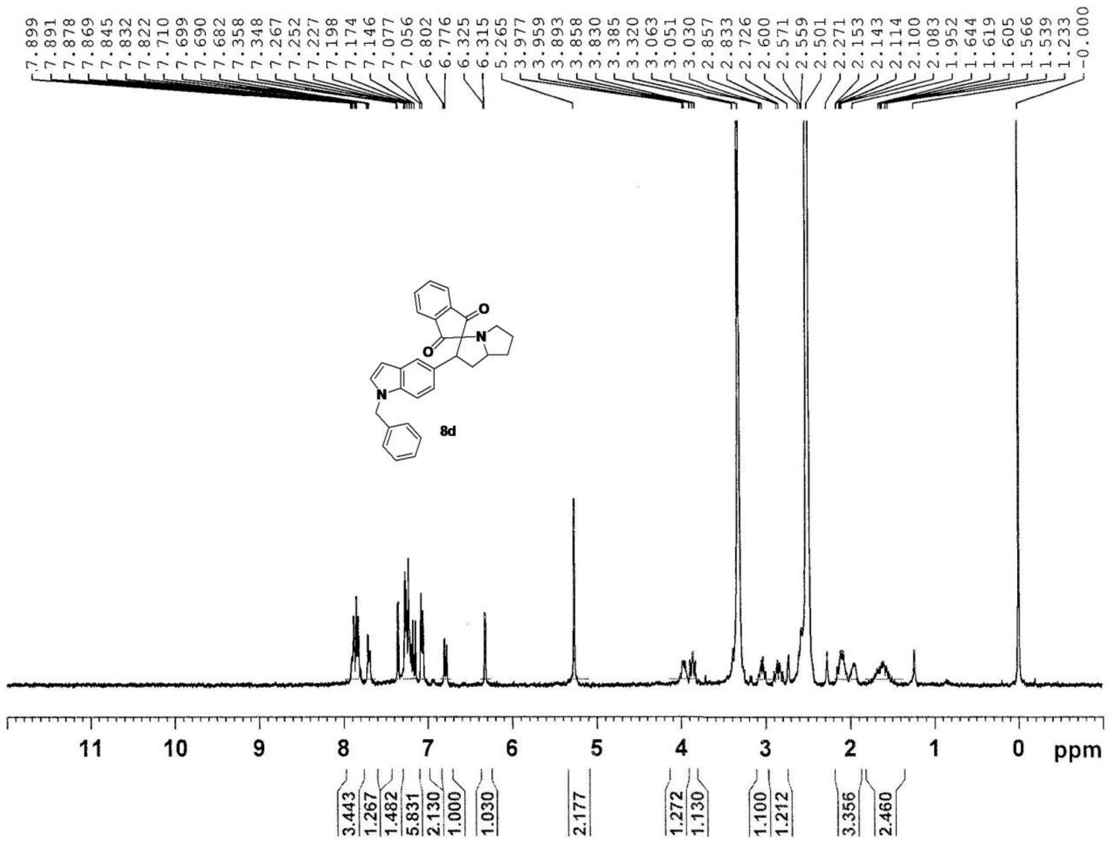

7.899
7.891
7.878
7.869
7.845
7.832
7.822
7.710
7.699
7.690
7.682
7.358
7.348
7.267
7.252
7.227
7.198
7.174
7.146
7.077
7.056
6.802
6.776
6.325
6.315
5.265
3.977
3.959
3.893
3.858
3.830
3.385
3.320
3.063
3.051
3.030
2.857
2.833
2.726
2.600
2.571
2.559
2.501
2.271
2.153
2.143
2.114
2.100
2.083
1.952
1.644
1.619
1.605
1.566
1.539
1.233
-0.000
O
O
N
N
8d
11
10
9
8
7
6
5
4
3
2
1
0
ppm
3.443
1.267
1.482
5.831
2.130
1.000
1.030
2.177
1.272
1.130
1.100
1.212
3.356
2.460

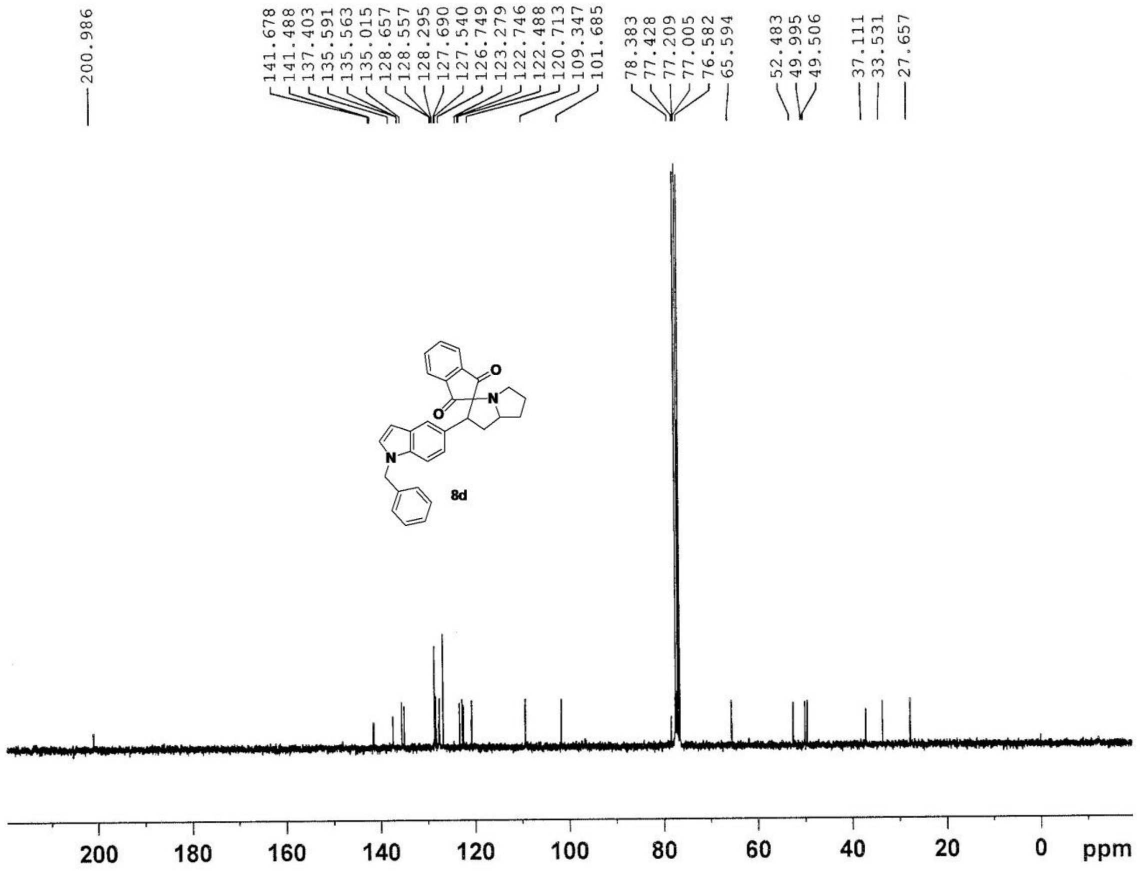

200.986
141.678
141.488
137.403
135.591
135.563
135.015
128.657
128.557
128.295
127.690
127.540
126.749
123.279
122.746
122.488
120.713
109.347
101.685
78.383
77.428
77.209
77.005
76.582
65.594
52.483
49.995
49.506
37.111
33.531
27.657
8d
200
180
160
140
120
100
80
60
40
20
0
ppm

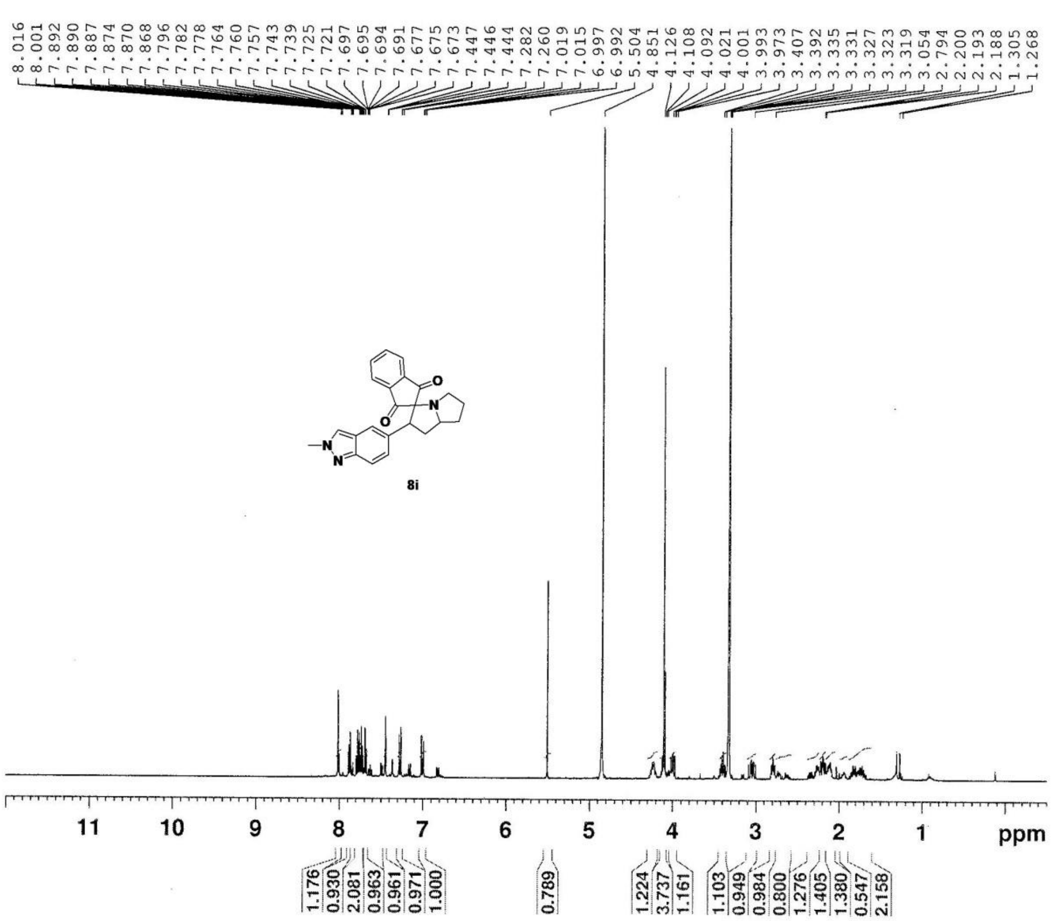

8i

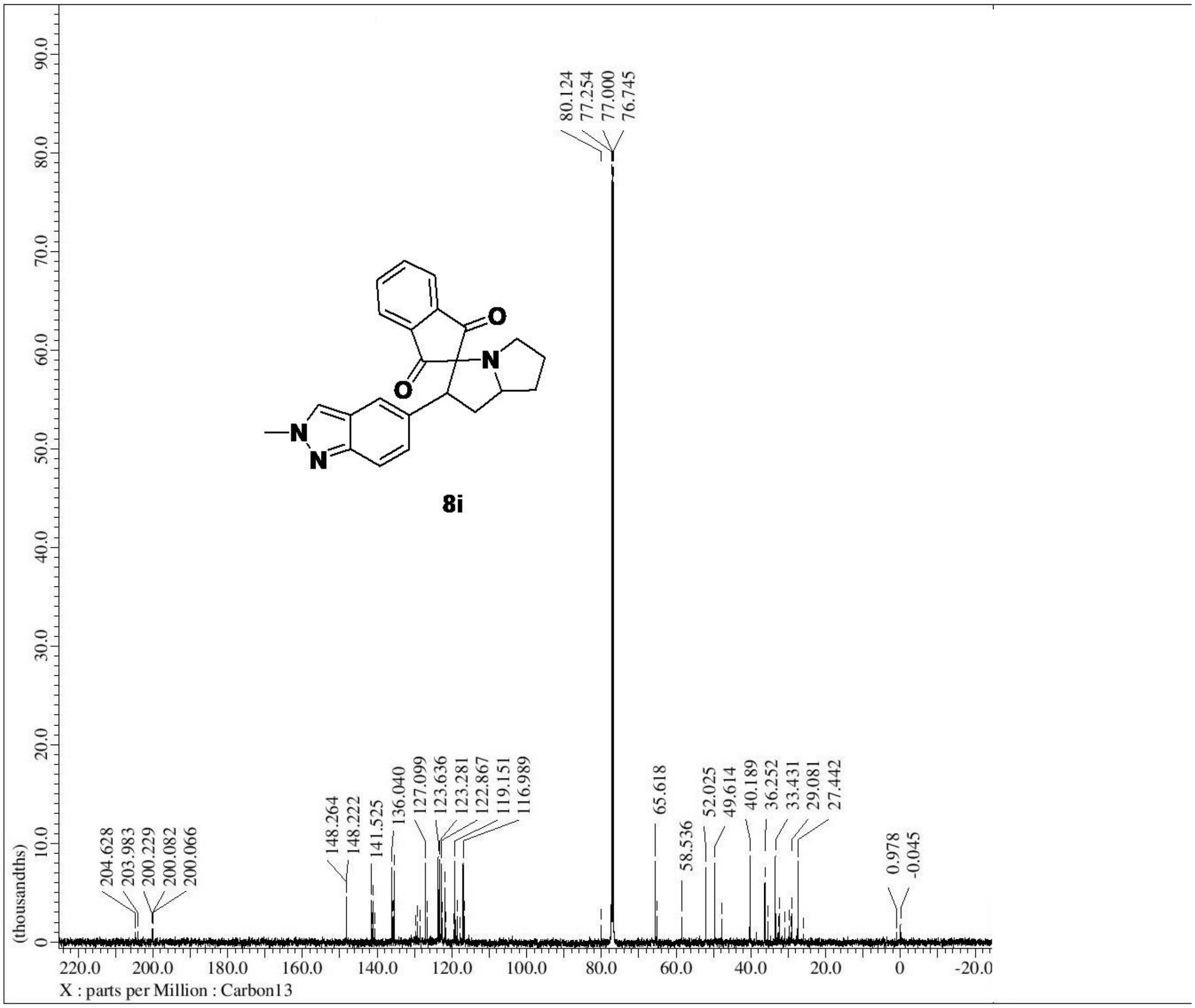
80.124
77.254
77.000
76.745
8i
204.628
203.983
200.229
200.082
200.066
148.264
148.222
141.525
136.040
127.099
123.636
123.281
122.867
119.151
116.989
65.618
58.536
52.025
49.614
40.189
36.252
33.431
29.081
27.442
0.978
-0.045
(thousandths)
X : parts per Million : Carbon13

X-ray Crystal structure determination of 7a and 7f

The X-ray diffraction data sets were collected on a Bruker SMART APEX II diffractometer using Mo Kα radiation. The crystal-to-detector distance was fixed at 40 mm for the crystal. The scan width per frame was $\Delta\omega = 0.5°$. The cell refinement and data reduction were carried out using the SAINTPLUS and numerical absorption correction using the analytical method was performed by face indexing. The sorting, scaling and merging were carried out by SORTAV. The crystal structures were solved by direct methods using SHELXS97 and refined in the spherical-atom approximation (based on F^2) by using SHELXL97 included in the WinGX package suite. The ORTEP diagrams were generated using POV−Ray. Supporting information for this paper contains crystallographic data and structure refinement of the title compounds.

Crystal Data for 7a

Table S1: Crystal data and structure refinement for **7a**

CCDC number	CCDC 1520097
Empirical formula	$C_{22}H_{20}N_2O_2$
Formula weight	344.40
Temperature	293 K
Wavelength	0.71073 Å
Crystal system, space group	Triclinic, P-1
a(Å), b(Å), c(Å)	7.5967 (16), 8.4114 (16), 14.507 (3)
α (°) β (°) γ(°)	98.389 (12), 94.880 (14), 95.230 (13)
Volume, Z	V= 909.0 (3), 2
Calculated density	1.258 g/m^3
Absorption coefficient	0.075 mm^{-1}
F(000)	365.0
Crystal size (mm^3)	0.16 x 0.21 x 0.24
Theta range for data collection	1.4 to 25°
Limiting indices	-9 ≤ h 17≥ l ≥ 17- ,9≥ k ≥ 9- ,9≥
Reflection collected/unique	2192/3069 [R_{int} =0.0448]
Completeness to theta	0.997
Refinement method	Full-matrix least-square on F^2
Data/restraints/parameters	3155/0/237
Goodness-of-fit on F^2	1.070
Final R indices [I>2σ(I)]	R1 = 0.0786,wR2 = 0.2166
R indices (all data)	R1 = 0.1008,wR2 = 0.2318
Largest diff. peak and hole	0.63 and -0.24 eÅ^{-3}

Table S2: Bond engths for **7a**

Bond	Length/Å	Bond	Length/Å
O1-C4	1.207(4)	C9-C10	1.409(4)
C1-C2	1.377(6)	C9-C13	1.422(5)
C1-C21	1.376(7)	C10-N11	1.377(4)
C2-C3	1.394(5)	C10-C16	1.386(4)
C3-C4	1.489(4)	N11-C12	1.448(4)
C3-C17	1.364(4)	N11-C14	1.371(4)
C4-C5	1.515(5)	C13-C14	1.363(5)
C5-C6	1.562(4)	C15-C16	1.370(5)
C5-C18	1.528(5)	C17-C18	1.507(5)
C5-N22	1.462(4)	C17-C20	1.380(4)
C6-C7	1.533(4)	C18-O19	1.210(4)
C6-C24	1.471(5)	C20-C21	1.376(6)
C7-C8	1.390(5)	N22-C23	1.435(5)
C7-C15	1.377(5)	N22-C25	1.456(4)
C8-C9	1.414(4)	C23-C24	1.523(5)

Table S3: Bond angles for **7a**

**Bond**	**Angle/°**	**Bond**	**Angle/°**
C21-C1-C2	121.0(4)	N11-C10-C9	108.5(3)
C1-C2-C3	117.8(4)	N11-C10-C16	130.3(3)
C2-C3-C4	128.4(3)	C16-C10-C9	121.3(3)
C17-C3-C2	121.1(3)	C10-N11-C12	126.0(3)
C17-C3-C4	110.5(3)	C14-N11-C10	108.3(3)
O1-C4-C3	125.3(3)	C14-N11-C12	125.8(3)
O1-C4-C5	125.9(3)	C14-C13-C9	108.0(3)
C3-C4-C5	108.8(2)	C13-C14-N11	109.5(3)
C4-C5-C6	111.2(3)	C16-C15-C7	123.2(3)
C4-C5-C18	102.3(2)	C15-C16-C10	118.3(3)
C18-C5-C6	113.3(2)	C3-C17-C18	108.7(3)
N22-C5-C4	114.4(2)	C3-C17-C20	120.8(3)
N22-C5-C6	100.4(2)	C20-C17-C18	130.5(3)
N22-C5-C18	115.8(3)	C17-C18-C5	108.8(2)
C7-C6-C5	116.8(3)	O19-C18-C5	125.9(3)
C24-C6-C5	101.6(3)	O19-C18-C17	125.2(3)
C24-C6-C7	120.5(3)	C21-C20-C17	118.6(4)
C8-C7-C6	123.3(3)	C1-C21-C20	120.8(4)
C15-C7-C6	117.9(3)	C23-N22-C5	109.0(3)
C15-C7-C8	118.8(3)	C23-N22-C25	116.4(3)
C7-C8-C9	120.3(3)	C25-N22-C5	116.6(3)
C8-C9-C13	136.0(3)	N22-C23-C24	105.5(3)
C10-C9-C8	118.2(3)	C6-C24-C23	105.5(3)
C10-C9-C13	105.8(3)		

Crystal Data for 7f

Table S4: Crystal data and structure refinement for **7f**

CCDC number	CCDC 1435212
Empirical formula	$C_{24}H_{25}N_3O_2$
Formula weight	387.47
Temperature/K	296.15
Crystal system	monoclinic
Space group	$P2_1/n$
a/Å	8.7531(18)
b/Å	17.051(3)
c/Å	14.480(3)
β/°	105.00(2)
Volume/Å^3	2087.5(7)
Z	4
ρ_{calc}g/cm^3	1.233
μ/mm^{-1}	0.080
F(000)	824.0
Radiation	MoKα (λ = 0.71073)
2Θ range for data collection/°	4.78 to 54.96
Index ranges	$-7 \leq h \leq 11$, $-18 \leq k \leq 22$, $-18 \leq l \leq 15$
Reflections collected	9013
Independent reflections	4669 [R_{int} = 0.0870, R_{sigma} = 0.1591]
Data/restraints/parameters	4669/0/265
Goodness-of-fit on F^2	0.975
Final R indexes [I>=2σ (I)]	R_1 = 0.1460, wR_2 = 0.3445
Final R indexes [all data]	R_1 = 0.3115, wR_2 = 0.4691
Largest diff. peak/hole / e Å^{-3}	0.31/-0.67

Table S5: Bond lengths for **7f**

**Bond**	**Length/Å**	**Bond**	**Length/Å**
O2-C8	1.204(7)	C15- C14	1.378(8)
C7-O1	1.251(7)	C15- C16	1.375(10)
C7-C5	1.447(10)	C17- C16	1.402(11)
C7-C9	1.534(9)	N2- C20	1.291(10)
C5-C4	1.375(9)	C14- C19	1.427(10)
C5-C6	1.361(10)	C14- C13	1.508(10)
N3-C9	1.447(9)	C16- C20	1.422(10)
N3-C11	1.452(10)	C4- C3	1.379(10)
N3-C10	1.465(9)	C22- C21	1.537(11)
C8-C9	1.543(11)	C22- C23	1.513(10)
C8-C4	1.507(10)	C22- C24	1.531(11)
C18-C17	1.406(9)	C13- C12	1.521(11)
C18-C19	1.354(10)	C12- C11	1.536(10)
N1-C17	1.385(9)	C3- C2	1.414(11)
N1-N2	1.368(8)	C2- C1	1.376(12)
N1-C21	1.453(9)	C6- C1	1.388(12)
C9-C13	1.577(9)		

Table S6: Bond angles for **7f**

**Bond**	**Angle/°**	**Bond**	**Angle/°**
O1-C7-C5	128.9(6)	C20- N2- N1	104.2(6)
O1-C7-C9	120.6(7)	C15- C14- C19	119.4(8)
C5-C7-C9	110.4(6)	C15- C14- C13	123.0(8)
C4-C5-C7	108.4(7)	C19- C14- C13	117.5(6)
C6-C5-C7	126.5(7)	C15- C16- C17	117.4(6)
C6-C5-C4	125.1(7)	C15- C16- C20	141.0(8)
C9-N3-C11	108.7(6)	C17- C16- C20	101.5(7)
C9-N3-C10	116.5(6)	C5- C4- C8	111.2(7)
C11-N3-C10	115.7(7)	C5- C4- C3	120.1(8)
O2-C8-C9	126.4(8)	C3- C4- C8	128.7(7)
O2-C8-C4	127.3(8)	C18- C19- C14	123.0(7)
C4-C8-C9	106.3(5)	C23- C22- C21	110.3(6)
C19-C18-C17	114.7(8)	C23- C22- C24	113.6(7)
C17-N1-C21	129.7(7)	C24- C22- C21	107.7(6)
N2-N1-C17	111.2(6)	C14- C13- C9	114.6(6)
N2-N1-C21	119.1(6)	C14- C13- C12	116.5(6)
C7-C9-C8	101.4(6)	C12- C13- C9	101.4(7)
C7-C9-C13	111.9(5)	C13- C12- C11	103.1(6)
N3-C9-C7	120.4(6)	N2- C20- C16	115.6(8)
N3-C9-C8	112.6(5)	C4- C3- C2	117.0(8)
N3-C9-C13	102.3(6)	N1- C21- C22	115.1(7)
C8-C9-C13	108.1(6)	C1- C2- C3	119.8(9)
C16-C15-C14	120.5(8)	C5- C6- C1	114.3(8)
N1-C17-C18	127.8(8)	N3- C11- C12	107.6(7)
N1-C17-C16	107.3(6)	C2- C1- C6	123.6(9)
C16-C17-C18	124.9(7)		
